# Multiregional Sequencing Analysis Reveals Extensive Genetic Heterogeneity in Gastric Tumors from Latinos

**DOI:** 10.1158/2767-9764.CRC-22-0149

**Published:** 2022-11-28

**Authors:** Ted W. Toal, Ana P. Estrada-Florez, Guadalupe M. Polanco-Echeverry, Ruta M. Sahasrabudhe, Paul C. Lott, John J. Suarez-Olaya, Alix A. Guevara-Tique, Sienna Rocha, Alexa Morales-Arana, Fabian Castro-Valencia, Shiro Urayama, Amanda Kirane, Dongguang Wei, Nora Rios-Sarabia, Rafael Medrano, Alejandra Mantilla, Magdalena Echeverry de Polanco, Javier Torres, Mabel E. Bohorquez-Lozano, Luis G. Carvajal-Carmona

**Affiliations:** 1 Genome Center, University of California, Davis, California.; 2 Grupo de Citogenética, Filogenia y Evolución de las Poblaciones, Universidad del Tolima, Ibagué, Colombia.; 3 UC Davis Comprehensive Cancer Center, Sacramento, California.; 4 Division of Gastroenterology & Hepatology, University of California, Davis, California.; 5 Department of Pathology and Laboratory Medicine, University of California, Davis, California.; 6 Unidad de Investigación en Enfermedades Infecciosas y Parasitarias, Unidad Médica de Alta Especialidad en Pediatría, Instituto Mexicano del Seguro Social, México City, México.; 7 Departamento de Sarcomas y Tubo Digestivo Alto, Unidad Medica de Alta Especialidad en Oncología Instituto Mexicano del Seguro Social (IMSS), México City, México.; 8 Departamento de Patología, Unidad Medica de Alta Especialidad en Oncología, Instituto Mexicano del Seguro Social (IMSS), México City, México.; 9 Department of Biochemistry and Molecular Medicine, School of Medicine, University of California, Davis, California.

## Abstract

Gastric cancer is a leading cause of cancer mortality and health disparities in Latinos. We evaluated gastric intratumoral heterogeneity using multiregional sequencing of >700 cancer genes in 115 tumor biopsies from 32 patients, 29 who were Latinos. Analyses focused on comparisons with The Cancer Genome Atlas (TCGA) and on mutation clonality, druggability, and signatures. We found that only approximately 30% of all mutations were clonal and that only 61% of the known TCGA gastric cancer drivers harbored clonal mutations. Multiple clonal mutations were found in new candidate gastric cancer drivers such as *EYS, FAT4, PCDHA1*, *RAD50, EXO1, RECQL4,* and *FSIP2.* The genomically stable (GS) molecular subtype, which has the worse prognosis, was identified in 48% of our Latino patients, a fraction that was >2.3-fold higher than in TCGA Asian and White patients. Only a third of all tumors harbored clonal pathogenic mutations in druggable genes, with most (93%) GS tumors lacking actionable clonal mutations. Mutation signature analyses revealed that, in microsatellite-stable (MSS) tumors, DNA repair mutations were common for both tumor initiation and progression, while tobacco, *POLE*, and inflammation signatures likely initiate carcinogenesis. MSS tumor progression was likely driven by aging- and aflatoxin-associated mutations, as these latter changes were usually nonclonal. In microsatellite-unstable tumors, nonclonal tobacco-associated mutations were common. Our study, therefore, contributed to advancing gastric cancer molecular diagnostics and suggests clonal status is important to understanding gastric tumorigenesis. Our findings of a higher frequency of a poor prognosis associated molecular subtype in Latinos and a possible new aflatoxin gastric cancer etiology also advance cancer disparities research.

**Significance:**

Our study contributes to advancing our knowledge of gastric carcinogenesis, diagnostics, and cancer health disparities.

## Introduction

Gastric cancer is the third cause of cancer mortality worldwide (1, 2). The disparity between gastric cancer incidence (∼1 M annual new cases) and mortality (∼760 K annual deaths) remains stubbornly minimal and little progress has been achieved toward treating advanced disease. This minimal disparity is partly explained by difficulties in early detection and the paucity of novel molecularly guided gastric cancer therapies. The Cancer Genome Atlas (TCGA) study showed that most gastric cancers harbor potentially “druggable” mutations (3). Interestingly, several studies have shown that extensive intratumoral heterogeneity (ITH) is present in most malignancies, which evolve by acquiring clonal and/or initiating mutations and subclonal and private progression mutations (4, 5). Understanding the clonal status of key tumor mutations is important to increase our knowledge of tumor evolution and for identifying the most promising and druggable targets. Most ITH studies have relied on bulk sequence analysis of multiple and spatially separated tumor biopsies (4, 5). This multiregional sequencing approach has advantages over single-biopsy-per-sample profiling. Studies have repeatedly shown that one-site/one-time biopsy sampling often misses a significant fraction of mutations (6). Single-biopsy TCGA studies showed that gastric cancers have a high mutation frequency and are likely to show extensive ITH (7). A study of heterogeneity in gastroesophageal adenocarcinoma found extensive mutational differences between primary tumors and metastatic lesions and significant discrepancies, potentially clinically relevant, at different sites within the primary tumor (8). ITH represents a significant challenge for target selection in precision medicine, as it likely explains the failure of most molecularly guided gastric cancer trials. Clonal mutations that drive tumorigenesis are widely considered the optimal drug targets, although nonclonal mutations can be useful targets, especially in combination therapies, if they play a functional role in subclones influencing tumor progression. The primary purpose of our study is to examine gastric cancer ITH patterns and evaluate its implication for tumor evolution and likely response to therapy, with the hypothesis that ITH would be present and help explain the difficulty of finding effective molecularly guided treatments. The study was enriched with patients of Latino ancestry, and we also explored ITH and gastric cancer genetic diversity implications for cancer health disparities in this population.

## Materials and Methods

### Patient Cohort

Our study cohort included 29 patients with gastric cancer of Latino ancestry (19 patients were recruited by Universidad del Tolima in Colombia, 9 by Instituto Mexicano del Seguro Social, in Mexico, and 1 by University of California, Davis in the United States) and 3 non-Latino Whites (recruited by University of California, Davis in the United States). Patient clinical information is shown in Table 1. Research protocols that were used to recruit human research subjects, who provided written informed consent, adhered to the Common Rule, and were approved by Institutional Review Boards from participating institutions in Colombia, Mexico, and the United States. Tumor biopsies were separated by >3 cm, and normal tissues were obtained from anatomically normal tissue identified during endoscopy or in surgical specimens. Biopsies were snap-frozen, and all patients had their tumors verified by a local surgical pathologist.

**TABLE 1:** Patient clinical and assigned molecular subtype information

Indiv	Type	Hpy	Ctry	Eth	Sex	Age	Hist	Anat	Stage
I_9709	EBV	N	Col	Lat	M	78	NA	B	IV
I_26163	EBV	N	Mex	Lat	M	72	I	B	IA
Subtotal	2	0							
I_10845	MSI	Y	Col	Lat	F	56	I	A	IV
I_8012	MSI	N	Col	Lat	M	62	I	C	IV
I_13137	MSI	N	USA	Wht	M	68	D	NA	IB
I_20447	MSI	N	USA	Wht	M	82	NA	C	NA
Subtotal	4	1							
I_13148	CIN	N	Col	Lat	F	37	D	A	IV
I_3755	CIN	N	Col	Lat	F	42	I	A	IIIB
I_7245	CIN	N	Col	Lat	F	64	I	A	IIIB
I_12808	CIN	N	Col	Lat	M	NA	NA	NA	IV
I_9278	CIN	N	Col	Lat	M	60	I	A	IV
I_9297	CIN	N	Col	Lat	M	60	M	C	IIIB
I_8164	CIN	N	Col	Lat	M	61	D	A	IIIB
I_9296	CIN	N	Col	Lat	M	67	M	A	IIB
I_26175	CIN	N	Mex	Lat	F	79	D	A/P	IIIC
I_26166	CIN	N	Mex	Lat	M	55	I	A/P	IIIB
I_26181	CIN	N	Mex	Lat	M	57	D	B/C	IIIC
I_13139	CIN	N	USA	Wht	F	74	D	A/P	NA
Subtotal	12	0							
I_9715	GS	Y	Col	Lat	F	31	I	L/A	IIIB
I_19344	GS	Y	Col	Lat	F	32	NA	NA	IV
I_11981	GS	Y	Col	Lat	F	49	D	L	IB
I_19343	GS	Y	Col	Lat	F	50	NA	NA	IV
I_6579	GS	N	Col	Lat	F	56	I	A	IV
I_9299	GS	N	Col	Lat	M	47	I	B/A	IIB
I_9710	GS	N	Col	Lat	M	53	I	A	II
I_14885	GS	N	Col	Lat	M	78	I	A	IIB
I_26169	GS	N	Mex	Lat	F	57	D	A/P	IIIC
I_26167	GS	N	Mex	Lat	F	65	M	B	IV
I_26180	GS	N	Mex	Lat	M	59	D	A/P	IIIA
I_26171	GS	N	Mex	Lat	M	71	D	B/A	IV
I_26172	GS	N	Mex	Lat	M	76	D	A/P	IIIA
I_13141	GS	Y	USA	Lat	M	55	NA	B	NA
Subtotal	14	5							
Total	32	6							

Abbreviations: A, antrum; Anat, Anatomic location; B, body; C, Cardia; CIN, Chromosomal instability; Col, Colombia; Ctry, Country; D, diffuse; EBV, Epstein-Barr virus; Eth, Ethnicity; F, Female; GS, Genomically stable; Hist, Histology; Hpy, *H. pylori* infection; I, intestinal; Indiv = Individual; L, lesser curvature; Lat, Latino; M, Male; M, mixed; Mex, Mexico; MSI, Microsatellite instability; NA, Not available; N, No; P, pylorus; Type, Molecular subtype; Wht, White; Y, Yes.

### Somatic Pan-cancer Panel Design

We selected 726 genes based upon known cancer risk (9), recurrence in previous gastric cancer samples, COSMIC Cancer Gene Census, TCGA Gastric cancer studies, and based upon expression list of cancer risk genes were collected from the literature (10–54), Color Genomics test suite (55), and DNA repair pathway genes (Supplementary Table S1) plus *TERT* promoter, microsatellite instable (MSI) regions, Epstein-Barr virus (EBV) and *Helicobacter pylori* sequences. Panel probes were designed using Agilent SureSelect XT2 Custom Capture technology (56) with a target capture region of 3.75 Mbp. There were 287 targeted genes that were covered less than 100%, and 24 of these were covered less than 90% (Supplementary Table S2).

### DNA Sequencing and Bioinformatics Pipelines

DNA samples were isolated with QIAGEN kits from 168 biopsies of gastric tumors and 37 normal gastric tissue samples adjacent to the tumors of 37 patients. Sequencing was performed on Illumina HiSeq 4000 (57) using paired-end 150 bp sequencing. Scythe (58) version 0.991 and Sickle (59) version 1.33 was utilized for trimming. Based upon DNA-seq quality control (QC), 15 biopsies and 3 patients were removed, gender mismatch QC led to six biopsies and 1 patient removed, and 115 tumor and 32 normal biopsies from 32 patients remained for the final analysis. Reads were aligned to GRCh38 using BWA (60) version 0.7.17. BROAD Institute Best Practices for Variant Calling with the GATK were followed (61). Germline single-nucleotide variants (SNV) were called using the joint variant caller multiSNV (62) version v2.3-15. Somatic variants were called using Mutect2 (63). Variants were annotated using Annovar suite (64). Additional germline and somatic variant filtering was applied as detailed in Supplementary Materials and Methods. A panel of normals was created with variants called in > = 2 samples. MSIsensor (65) was used to predict tumor sample MSI.

A clonality was assigned to each SNV: PRIVATE (only one tumor is a somatic variant), SUBCLONAL (greater than 1 but not all biopsies carry the variant), CLONAL (all tumors have variant), or NONE (applied to aberrant cases). PureCN (66), version 1.16.0 was used to perform copy-number variation (CNV), purity, and ploidy analysis, and biopsy mutation rate estimation (see Supplementary Materials and Methods for more details). Mappability files were created using GEM library (67) version 1.778 beta. Each tumor biopsy's mutation rate was estimated with PureCN. To address possible pseudoheterogeneity due to factors such as allele-specific imbalance or heterogeneous amplification, an in-house heuristic forced variant calling algorithm was used to look for mutant allele reads at levels too low to be called mutant when the same locus was called mutant in a sister biopsy. OncoKB gene list (68, 69) and FDA gastric cancer–targeted therapy genes (70) were combined, leaving 58 druggable genes overlapping our gene targets. Mutation signatures were estimated with deconstructSigs (71). A subset of the V3.2 signatures were used, retaining those deemed relevant to gastric cancer.

### Statistical Analysis

The statistical tests used in this study are Student *t* test for comparing sample means and Fisher exact test for comparing proportions in multiple categories. This study analyzes data from 32 patients and many more biopsies, so the data from patients and biopsies is not independent. The patient count is too low to provide substantial statistical power, so *P* values are used only when testing individuals independently of biopsies and the study is otherwise more descriptive in nature.

A detailed description of the methods for data processing and the software used for analysis is available in the Supplementary Materials and Methods file.

### Data Availability Statement

The data generated in this study were deposited in the European Genome-phenome Archive (Study ID: EGAS00001006650, Dataset ID: EGAD00001009622).

## Results

### Patient Characteristics

Table 1 shows the main characteristics of the patients analyzed in the study. None of these patients carried germline mutations in Lynch syndrome or hereditary diffuse gastric cancer genes (not shown). Mexican patients had a larger fraction of tumors with diffuse histology (Table 1). Colombian patients, on the other hand, were significantly younger than those from Mexico or United States (55 vs. 66 years, *P* = 0.020; Student *t* test). Colombian and Mexican patients had a similar distribution of molecular subtypes. Compared with available TCGA clinical data, our patient population has a similar sex distribution (Table 1; Supplementary Table S3), with 59% of our study patients being male versus 62% in TCGA. Our patients were however younger than those from TCGA (60 vs. 66 years, *P* = 0.0183; Table 1; ref. 3) and had a higher rate of advanced tumors (76% vs. 45%; Table 1; ref. 3).

### Gastric Tumor Genomic Landscape

**FIGURE 1:**
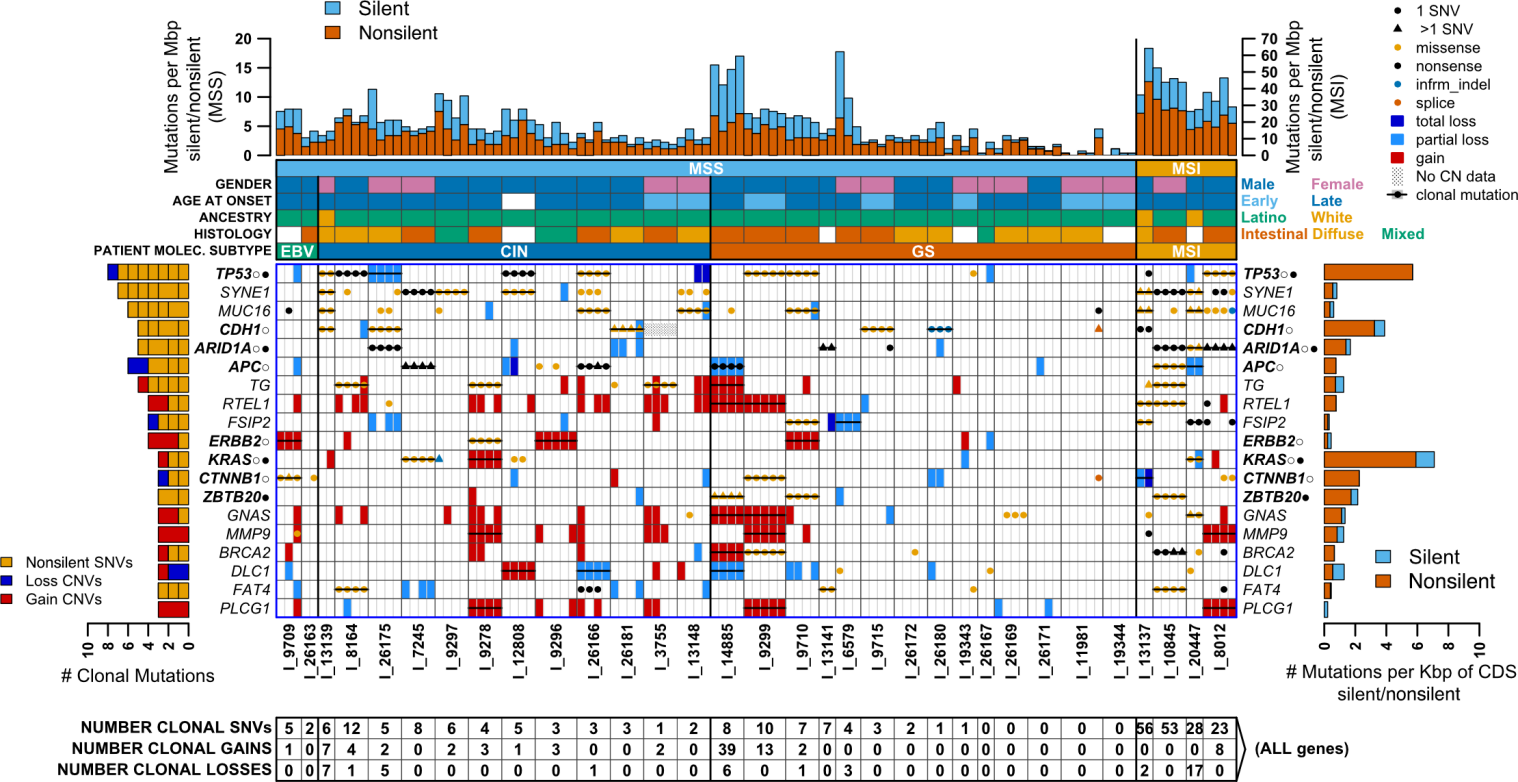
Genes clonally mutated in 3 or more gastric cancer MSS/MSI patients. Gene names shown on both left and right sides are those where at least 3 patients have clonal somatic nonsilent single-nucleotide or short indel (SNV), or copy-number gain/loss (CNV) mutations. Patients (I_#### IDs) are separated by a dark vertical line and patient biopsies within them are separated by a lighter line. SNV and CNV mutations are shown for each gene/biopsy combination. Gains and losses are distinguished by box color, while SNVs are shown as a filled circle or triangle using color to distinguish the mutation type (key at top right; nonsense mutations include stop gain/loss and frameshift). A triangle rather than a circle means a gene has more than one mutation in a patient, and the type shown is the one generally most detrimental. A bar through all biopsies means the mutation is clonal. The left-side bar plot shows the total number of clonal mutations of each type in each gene, with short vertical lines separating individual patients. Numbers in boxes at the bottom give the total number of clonal mutations of each type in each patient in *all genes* (not just the ones shown). Patient molecular subtypes are indicated by color-coded bars just above the main figure area, and above those are additional color-coded bars indicating patient microsatellite instability status (MSS/MSI), sex, age of onset of gastric cancer (where late onset was defined as occurring at age >50), ancestry (Latino or White), and tumor histology. The top bar plot shows the SNV mutation rate in each biopsy, in the region targeted by the panel, with nonsilent and silent rates distinguished by color. MSS patients have a lower mutation rate and use the *y*-axis scale on the left, while MSI patients with a higher rate use the right-side scale. The right-side bar plot shows the SNV mutation count in each gene summed over all 115 biopsies, normalized by dividing by the gene CDS length in Kbp, with nonsilent and silent mutations distinguished by color. Gene names are in bold when they were found to be gastric cancer drivers by TCGA gastric cancer study, with open circles denoting driver genes in non-hypermutated (MSS) TCGA samples and filled circles denoting those in hypermutated (MSI) samples.

We evaluated 115 tumor biopsies from 32 patients (Table 1) using our cancer panel (Supplementary Data S1). On average, each tumor biopsy was sequenced at 336X depth and each normal biopsy at 225X (Supplementary Data S2). After filtering out likely false positives, we identified a total of 1,319 different somatic mutations (mean 41/patient), or 1,326 counting recurrences in different patients, or 2,594 counting occurrences in each individual tumor biopsy (mean 23/biopsy), in 473 genes. These included 771 nonsilent coding mutations (635 SNVs and 136 indels; mean 24/patient), or 775 total across patients including recurrences, or 1,543 counting occurrences in each individual tumor biopsy (mean 13/biopsy) occurring in 355 panel-targeted genes (Fig. 1; Supplementary Figs. S1–S8; Supplementary Data S3). For TCGA molecular subtypes, of the 32 patients, 2 were classified as EBV (6.2%), 4 as MSI (12.5%), 12 as chromosomal instability (CIN) (37.5%), and 14 as GS (43.8%). When analyses were restricted to Latinos (*n* = 29 patients), subtype frequencies were 38% for CIN, 7% for EBV, 48% for GS, and 7% for MSI (Supplementary Table S3), and when our Mexican and Colombian Latino subtypes were compared, there were twice as many Colombian CIN and GS patients (*n* = 16) as Mexican (*n* = 8) but the overall difference in subtype proportions was not significant by Fisher exact test (*P* = 0.8). The subtype frequencies in our Latino patients were significantly different from those in all TCGA patients (*P* = 0.033; Fisher exact test). Within TCGA's White and Asian patients (*n* = 238 White, *n* = 74 Asian), the rate was similar to ours for EBV (8% for Whites, 11% for Asians), higher for MSI (18% for Whites, 20% for Asians) and CIN (61% for Whites, 51% for Asians), and much lower for GS (13% for Whites, 18% for Asians).

### Somatic Mutation and Phylogenetic Analyses Showed Varying ITH

We next analyzed the distribution of SNVs, indels, and CNVs in the different biopsies of each patient and classified each mutation as clonal (occurring in all individual tumor biopsies from the same patient), subclonal (occurring in more than one but not all individual tumor biopsies), or private (occurring in one individual tumor biopsy only). Supplementary Data S3 shows counts of all somatic SNVs/indels and their clonal status. Of the 1,326 such mutations, 428 were clonal (32%, mean 13/patient), 213 were subclonal (16%, mean 7/patient), and 685 were private (52%, mean 21/patient). The clonal SNV fraction in patients of different molecular subtypes was about the same in GS and CIN (27%; GS mean 5.9/patient; CIN mean 6.9/patient), higher in EBV (34%, mean 8.5/patient), and MSI (36%, mean 61.5/patient). There was at least one clonally mutated gene in 27 of the 32 patients (all 5 patients without clonal changes had the GS subtype). In contrast, nonclonally mutated genes were found in all but 1 patient (Fig. 1; Supplementary Fig. S1). Phylogenetic trees of clonal and nonclonal changes were generated to visualize the evolution of each tumor (Supplementary Fig. S2). These trees graphically illustrate the branched evolution pattern followed by gastric cancers.

### Clonally Mutated Genes in Microsatellite-Stable and MSI Patients

In our 28 microsatellite-stable (MSS) patients, we found 15 genes with clonal nonsilent SNVs/indels in multiple patients. Of these, one gene was clonally mutated in 6 patients (*TP53*), one in 5 patients (*CDH1*), two in 4 patients (*MUC16, SYNE1*), and two in 3 patients (*APC, TG*; Fig. 1; Supplementary Figs. S1 and S3). In addition, even though our targeted approach had limitations to accurately call CNVs, our analyses identified 12 genes that were clonally amplified or deleted in multiple MSS patients, including two with a mixture of amplifications and deletions (*DLC1*, *WRN*; Supplementary Fig. S4), one that was clonally amplified in 3 patients (*ERBB2*), and nine that were clonally amplified in 2 patients each (*FGFR2*, *KLF5,* the 8q24 *MYC* and *RECQL4* genes, and the 20q12-13 genes *PLCG1*, *MMP9*, *GNAS*, *LAMA5,* and *RTEL1,*Supplementary Figs. S4–S6). Counting both nonsilent SNVs/indels and CNVs, a total of 39 genes were clonally altered in multiple MSS patients. Of those, nine are known TCGA gastric cancer drivers (*APC*, *CDH1*, *CTNNB1*, *ERRB2*, *KRAS*, *RNF43, ARID1A,* and *TP53* in MSS tumors, and *ZBTB20* in MSI tumors), eight have been identified as gastric cancer drivers in non-TCGA studies (*MUC16, DLC1, MMP9, FASN, LAMA5, EGFR*, *BRCA2,* and *FGFR2*), 14 have been identified as drivers for other cancer types by TCGA (*ATAD2*, *ATR*, *CSDE1*, *CSMD3*, *ELF3*, *ERBB4*, *KLF5, TRPA1*, *TSHZ2*, and *PLCG1*) and non-TCGA studies (*GNAS*, *MYC*, *SYNE1*, and *TG*), and eight have not been previously identified as gastric cancer drivers (*EYS, FAT4, FSIP2, PCDHA1, RAD50, RECQL4, RTEL1,* and *WRN*). These latter clonally mutated genes should be considered candidate gastric cancer driver genes for future studies.

In our 4 MSI patients, we found 17 genes with clonal nonsilent SNV/indels in multiple patients, including 2 in 3 patients (*ARID1A, SYNE1*) and 15 in 2 patients (*ALPK2, ATM, CDC27, CDK12, ESR1, EXO1, FSIP2, KMT2E, LRRK2, MACF1, MUC16, NEB, PIK3CA, RNF111,* and *RTEL1*; Fig. 1; Supplementary Fig. S1 and S5). Five of these genes are known TCGA drivers (*ARID1A, ALPK2, PIK3CA,* and *RNF111* in MSI tumors and *MACF1* in MSS tumors*),* one has been identified as a gastric cancer driver in non-TCGA studies (*MUC16*), eight are known drivers for other cancer types in TCGA (*ATM*, *CDC27, CDK12, ESR1, KMT2E,* and *LRRK2*) and non-TCGA studies (*SYNE1* and *NEB*), and three have not been previously identified as drivers (*EXO1, RTEL1,* and *FSIP2*). These three should also be considered candidate driver genes for future studies.

It is always possible for clonal mutations to be passengers rather than drivers, more so the larger the gene. Using the definition of long gene length as above the 75% of all genes in our panel, we examined the long clonally mutated genes mentioned above for evidence of likely pathogenic changes [defined as a loss-of-function mutation, a known cancer hotspot mutation, annotated as pathogenic in ClinVar (72), amplification of a known oncogene, or complete deletion of the entire gene or its wild-type allele]. Because of the absence of clonal mutations classified as likely pathogenic, nine genes (*TG, WRN, PLCG1, LAMA5, RTEL1, FASN, CDK12, LRRK2,* and *MACF1*), were considered unlikely to be candidate gastric cancer drivers. Conversely, in five of these genes, *all* clonally mutated patients had mutations that were likely pathogenic (*CTNNB1, ELF3, ATM, KMT2E,* and *PIK3CA*), lending stronger support to their candidate gastric cancer driver status.

Nonclonally mutated genes are summarized in Supplementary Fig. S7. Genes with a relatively high nonclonal mutation frequency include *STK11*, *GPS2*, *NDUFB9*, and *AXIN2*. The tumorigenesis role, particularly in cancer progression, of these nonclonally mutated genes should be evaluated in future cancer biology studies.

### The Known TCGA Gastric Cancer Drivers are Clonally Heterogeneous

**FIGURE 2:**
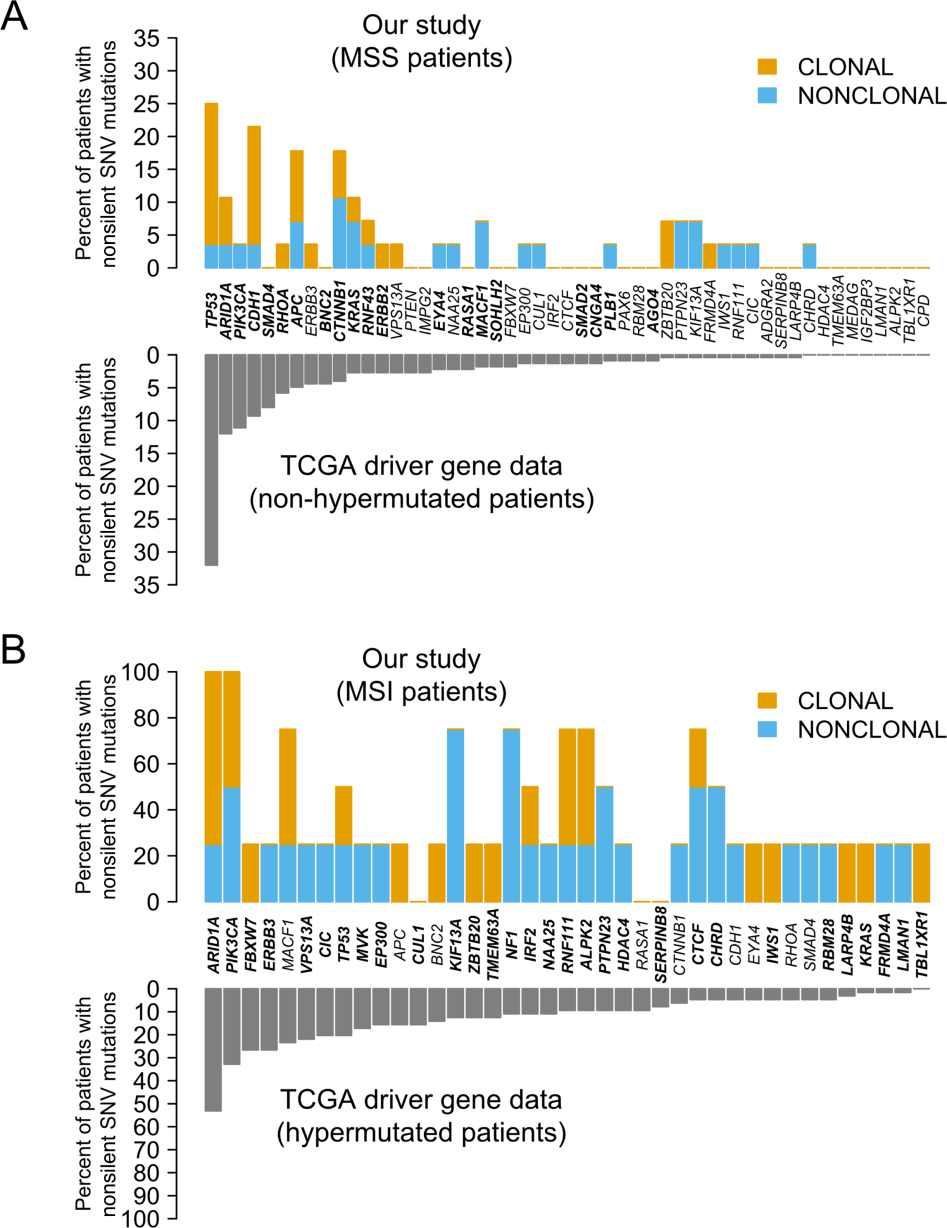
Comparison of TCGA gastric cancer driver mutation frequency with this study. **A,** Genes significantly mutated in non-hypermutated TCGA samples, compared to MSS patients in our study. **B,** Similarly, for hypermutated TCGA samples compared with our MSI patients.

Our panel contained 54 well-covered genes that were identified as gastric cancer drivers by TCGA. We found that 11 of these genes harbored nonsilent clonal SNV/indel mutations in multiple patients (*TP53, CDH1, ARID1A, APC, KRAS, CTNNB1, ZBTB20, PIK3CA, RNF111, ALPK2,* and *MACF1*; Fig. 1; Supplementary Fig. S1). In addition, 20 known gastric cancer TCGA drivers had clonal SNV mutations in 1 patient each in our study (Supplementary Fig. S1). Figure 2 contrasts the number of patients having clonal and nonclonal mutations in these known drivers in our study with the number of patients with mutations in TCGA. Most known drivers had both clonal and nonclonal mutations, with half of them clonally mutated in at least 1 patient while the other half was either nonmutated or only nonclonally mutated. Of note, *ZBTB20*, a driver identified by TCGA in MSI tumors, was clonally mutated only, in multiple patients. In contrast, *CIC*, *NF1*, *KIF13A*, *PTPN23,* and *CHRD* (all MSI drivers) were mutated in multiple patients, but always nonclonally. Indeed, after *TP53* and *ARID1A* (clonally mutated in 6 patients each), *CDH1* and *APC* (clonally mutated in 5 patients each), and *ERBB2* (clonally mutated/amplified in 4 patients), *ZBTB20, KRAS*, and *BRCA2* were the fourth most common clonally mutated known driver genes in our study. When these analyses were stratified by the patient's country of origin or age of gastric cancer diagnosis (≤50 y vs. >50 y), we found no significant difference in our study. Overall, our clonal analyses of known TCGA drivers support the notion that the driver status of some of these genes is worthy of reexamination in larger ITH studies. Alternatively, as our sample was enriched with tumors from Latino patients, these differences may reflect population differences, as TCGA included mostly Whites in their analyses.

### ITH in Druggable Genes

Our panel included 58 druggable genes. One-third (*n* = 11, 34%) of our patients had at least one clonal likely pathogenic change (defined above) in a druggable gene. Clonal likely pathogenic changes in druggable genes were found in all four MSI patients (100%), 5 of the 12 patients with CIN (42%), 1 of the 4 EBV patients (25%), and only 1 of our 14 GS patients (7%). The pathways with the highest number of clonal likely pathogenic changes included tyrosine kinase receptors (*ERBB2*, *EGFR*, *FGFR2*, and *FLT4*, mutated in 4 patients), homologous recombination repair (*BRCA2, ATM,* and *ATR*, mutated in 4 patients), PI3K/AKT/MTOR (*PIK3CA* and *AKT1*, mutated in 3 patients), and RAS/RAF/MAPK (*KRAS*, mutated in 3 patients). Finally, we found six genes with nonclonal pathogenic mutations in 2 or more patients (*EGFR, ERBB2, KRAS, NF1, PIK3CA,* and *STK11;*Supplementary Figs. S7 and S8).

### Mutation Signatures in MSS Tumors

We were also interested in examining whether different processes could mediate clonal and nonclonal mutations (Supplementary Table S4; Supplementary Figs. S5, S8, and S9). As most of our patients had MSS tumors (28/32), we focused on initial analyses in this group. MSS clonal mutations mainly resulted from signatures SBS1 (deamination), SBS3 (homologous recombination deficiency, HRd), SBS4 (tobacco), SBS5 (age), and SBS10b (*POLE* mutations, *POLE*m). Nonclonal mutations, on the other hand, had signatures associated with HRd, tobacco, age, mismatch repair deficiency (MMRd, SBS6, and SBS15), reactive oxygen species damage (ROSd, SBS18), and aflatoxin exposure (SBS24, aflatoxins). As our study was enriched with Latino patients, we compared their signatures with those in TCGA MSS patients (Supplementary Table S4; Supplementary Fig. S9). Deamination and age signatures were found in Latinos and TCGA; however, HRd-, SBS6/MMRd-, ROSd-, and aflatoxins-associated signatures were present only in Latinos.

We also analyzed signatures in CIN and GS tumors separately (Supplementary Table S4; Supplementary Fig. S9). The HRd signature was found in both CIN and GS clonal mutations but not in nonclonal mutations. The *POLE*m signature was found in clonal mutations of GS but not CIN patients. The SBS15/MMRd was found in both CIN and GS but only in nonclonal mutations. These findings suggest that CIN and GS tumors may result from different mutational processes involved in tumor initiation and progression.

Finally, as GS was our most common subtype, we explored associations between GS tumor signatures and histology in our study and TCGA (Supplementary Table S4). Our study's GS diffuse and intestinal tumor mutations had the age, SBS15/MMRd, and aflatoxins signatures. Age, but not the SBS15/MMRd or aflatoxin signatures, was also detected in TCGA GS diffuse and intestinal tumors. In our study and TCGA, GS diffuse mutations resulted from deamination and *POLE*m signatures. In GS intestinal tumors, we found SBS6/MMRd in our Latinos and TCGA, while HRd and ROSd were exclusively found in our study. These findings suggest that different mutational processes may affect histologic types in GS tumors.

### Mutation Signatures in MSI Tumors

Our study only included 4 MSI patients, so we mainly evaluated mutation signature differences by clonal status. Clonal mutations in MSI tumors were primarily the result of MMRd (SBS15 and SBS26) and deamination. Nonclonal mutations, on the other hand, had signatures associated with deamination, tobacco, and MMRd (SBS6, SBS15, SBS21, and SBS26). These findings suggest, as expected, that MMRd is required for MSI tumor initiation, while tobacco may be important for MSI tumor progression.

## Discussion

This study represents a comprehensive investigation of ITH in gastric cancer, a worldwide leading cause of cancer incidence and death (2). Our study showed that gastric cancers are characterized by a complex genetic architecture and suggested the existence of novel driver genes. Our druggable target analysis identified key pathways and genes that often harbor clonal mutations, and that should be prioritized for therapeutic development. Mutational signature analyses suggested that carcinogens likely play a different role in clonal and nonclonal mutations, across molecular and histologic subtypes and that population-specific exposures, such as aflatoxins, may also influence gastric cancer etiology. Therefore, our study reports findings important to understanding gastric cancer etiology, disparities, tumor evolution, and future therapeutic development.

TGCA studies have demonstrated that gastric cancers are among the most genetically diverse tumors (7), with each gastric cancer harboring approximately 500 coding mutations (3). The mutation rate, however, varies greatly between molecular subtypes, with MSI tumors having the highest number of alterations and GS tumors the lowest (3). Consistent with previous work (3), our MSI tumors harbored the highest number of both clonal and nonclonal mutations, followed by CIN, EBV, and GS tumors. These differences in mutation rate are important not only for understanding tumor evolution but also for making the best-informed choice of targeted therapies or immunotherapies. Interestingly, mutation patterns in our study highlighted several important findings about gastric cancer drivers. First, it became evident that the list of gastric cancer driver genes is likely larger than that reported by TGCA. Our results suggested that the clonal mutation status in both known and potentially new candidates is important and should be considered to help validate their “driver” status. In our analyses of known TCGA gastric cancer drivers, for instance, we showed that only approximately 60% had clonal mutations, raising questions about the initiation versus progression “driver” status of genes such as *CIC*, *NF1*, *KIF13A*, *PTPN23,* and *CHRD*, which were mutated in multiple patients but always nonclonally. Even though clonal mutations may be the obvious targets for therapies, it is possible that some nonclonal mutations play an important role in cancer progression. Our findings are therefore intriguing and suggest that larger ITH studies should evaluate whether these nonclonally mutated genes are indeed drivers and whether they are involved in tumor progression or only harbor passenger and/or neutral mutations.

An interesting aspect of our analyses is that we identified several recurrent clonally mutated genes. Many of them, such as *ATAD2, ATR, BRCA2, CSDE1, CSMD3, DLC1, EGFR, ELF3, ERBB4*, *FGFR2, KLF5, TRPA1*, *TSHZ2*, *GNAS, MYC,* and *MMP9* for *MSS* tumors and *ATM, CDC27, ESR1, KMT2E,* and *NEB* for MSI tumors, have been previously identified as drivers by TCGA/non-TCGA studies for other cancer types but not for gastric cancer. We also found six genes in MSS tumors (*EYS, FAT4, FSIP2, PCDHA1, RAD50,* and *RECQL4*) and two in MSI tumors (*EXO1* and *FSIP2*) that were clonally mutated in multiple patients and have not been previously identified as drivers of gastric cancer or other cancers. Interestingly, many of these new genes are involved in key processes disrupted in gastric tumorigenesis, such as extracellular matrix (*EYS*) and cell adhesion (*FAT4* and *PCDHA1,* which are protocadherins) or homologous recombination repair (*RAD50* and *RECQL4*). These potentially new gastric cancer driver genes, as well as genes with high nonclonal mutation frequency, such as *STK11*, *GPS2*, *NDUFB9*, and *AXIN2,* represent good candidates for inclusion in future studies of gastric tumorigenesis.

We and others have shown that genes involved in homologous recombination repair are important in both gastric cancer risk and tumorigenesis (73–75), which is consistent with our observation of multiple patients with clonal nonsilent mutations in *ATR*, *ATM,* and *BRCA2* and with the mostly clonal nature of the HRd-associated mutation*.* Our study also found many patients with clonal pathogenic mutations in the RAS/RAF pathway gene *KRAS* and the PI3K/MTOR/AKT pathway gene *PIK3CA,* providing further evidence of their importance in gastric cancer biology. While *ERBB2* and *ERBB3* are both molecular targets of FDA-approved gastric cancer therapies and are known TCGA gastric cancer drivers (3, 76), the high number of patients with mutations in other related tyrosine kinase genes (*FGFR2*, *EGFR/ERBB1*, and *ERBB4)* suggest that these should also be considered important genes in gastric cancer biology. These results suggest clonal status can further identify novel genes that are important in gastric tumor biology.

Gastric cancer targeted therapies have been notorious for their failure in late-stage trials, with only two (targeting *ERBB2* and *VEGFR2*) currently approved by the FDA (76–78). While gastric cancers are characterized by one of the highest mutation rates among all solid malignancies, and TCGA suggested that approximately 70% of them harbor potentially actionable or druggable mutations (3), our ITH analyses indicated that even though a significant fraction of gastric cancers do indeed carry druggable mutations, only about 60% of these tumors have clonal mutations in druggable genes. However, we also found that clonal druggable mutations are closely associated with the molecular subtype, with the good-prognosis MSI subtype having the highest number of druggable targets and the poor-prognosis GS subtype having the lowest (79). Indeed, all 4 MSI patients in our study harbored multiple clonal actionable mutations, while >90% of GS tumors lacked such mutations. This suggests that MSI tumors will be highly amenable to targetable therapy development, which will likely be beneficial for future combinations with immunotherapies (80). GS tumors, on the other hand, remain a significant challenge in drug development, and future efforts should focus on identifying additional targets (such as methylation or synthetically lethal combinations) for preclinical studies and clinical trials. As GS tumors seem more prevalent in Latinos, such studies should involve race and/or ethnic appropriate models and participants (81).

Our mutation signature analyses revealed interesting differences between MSS and MSI tumors and between Latino and TCGA tumors. In MSI tumors, MMRd and deamination likely drive tumor initiation while tumor progression seems to be driven by tobacco-associated mutations. While an association between the MMRd mutation signature and MSI tumorigenesis makes sense, the fact that tobacco may accelerate MSI tumor progression is consistent with previous studies showing a stronger association with gastrointestinal MSI tumors in heavy smokers (82–84) and with the fact that Lynch syndrome patients carrying germline MMR mutations are particularly susceptible to tobacco carcinogens (85). Our signature analyses in MSS tumors, on the other hand, revealed that deamination- and *POLE*m-associated mutations likely drive tumor initiation; HRd-, tobacco- and age-associated mutations influence both tumor initiation and progression; and MMRd-, ROSd- and aflatoxin-associated mutations play a role in tumor progression. Our separate analyses of CIN and GS tumors revealed that *POLE*m/SBS10b seems to primarily play a role in GS tumor initiation. Our histologic comparison within GS tumors also revealed consistent associations, in our study and in TCGA, of the importance of deamination in diffuse histology and of MMRd in intestinal histology. Finally, our finding of an aflatoxin mutational signature is also novel and intriguing and suggests that larger and further studies should examine the role of this risk factor (which has been found in Latinos with liver and gallbladder cancers) in gastric tumorigenesis (86–88). This finding, if replicated, also highlights the benefits of racial and/or ethnic diversity in cancer genetics studies.

Gastric tumors are one of the leading causes of cancer health disparities (89, 90). As our study was enriched with Latino patients, our results are important to advance precision health equity in this population. Future studies should evaluate many of the novel findings found in our study and assess whether such patterns are more common in Latinos or are also observed in other populations. Some of our results are particularly puzzling, such as our significant difference in molecular subtypes when compared with TCGA, with our study having a significantly higher prevalence of GS tumors, which are known to have the poorest prognosis in gastric cancer (79) and which are more commonly associated with diffuse histology (3). A high frequency of GS tumors in Latinos, also recently reported in Texas (91), therefore may explain some of the observed disparities in Latino gastric cancer outcomes, and the development of future therapies for this subtype should be a priority in gastric cancer disparity research.

Our observational study however has some limitations. First, it was solely focused on DNA sequence changes in a panel of cancer genes. Genome-wide or exome-wide analyses would likely have led to improved signature analyses. While our study did not have the ability to evaluate methylation or gene expression ITH, our findings showed a striking level of genetic complexity in gastric cancers, which may explain why these tumors are so difficult to treat. We also hope that future functional studies investigate the tumorigenesis role of some of the putative new drivers highlighted in our study. Furthermore, our study was enriched with advanced tumors, which could introduce biases compared with TCGA. Despite these limitations, we believe that our study has several strengths, focused on an understudied population, and reporting several novel findings that will likely contribute to advancing gastric carcinogenesis and disparities.

In sum, we carried out a comprehensive evaluation of gastric tumor genetic diversity. Our study found that Latinos are enriched with a poor prognosis and chemotherapy-resistant subtype that likely account for some of the outcome disparities experienced by Latinos. Our findings showed a striking level of genetic complexity in gastric cancers, explaining why these tumors are so difficult to treat. We hope our results help advance target selection for gastric cancer therapies and aid in understanding gastric tumorigenesis and disparities.

## Supplementary Material

Supplementary Data File S1 Spreadsheet of pan-cancer panel gene composition, with citation codes

Supplementary Data File S2 Spreadsheet of sample mean sequencing depths

Supplementary Data File S3 Spreadsheet of counts of patients with SNV/indel mutations in each gene

Supplementary Materials and Methods, Tables and Figures SM1 PDF file of supplementary methods and materials, and supplementary figures and tables
