# Supplementary material for: Multiregional Sequencing Analysis Reveals Extensive Genetic Heterogeneity in Gastric Tumors from Latinos
Source: Cancer Res Commun. 2022 Nov 28;2(11):1487–96. doi: 10.1158/2767-9764.CRC-22-0149 (PMC10035402; doi:10.1158/2767-9764.CRC-22-0149)
Supplement: Supplementary Materials and Methods, Tables and Figures SM1 — PDF file of supplementary methods and materials, and supplementary figures and tables [file crc-22-0149-s04.pdf]

Multi-regional Sequencing Analysis Reveals Extensive Genetic Heterogeneity in Gastric Tumors from Latinos

Supplementary material

Supplementary figures and files referred to in the manuscript 3
Somatic pan-cancer panel design 3
Anonymous Patient and Sample IDs 3
DNA extraction and sequencing 3
R packages used 3
Reference genome and reference BED file construction 4
Project BED file construction 4
Read filtering and mapping 4
Quality control testing for artifacts, cross-sample contamination, and gender detection 4
Extraction of unaligned and Epstein-Barr and H. pylori reads 5
MSI assessment 5
Variant calling 5
Panel of Normals construction 5
SNV variant calling with multiSNV 5
Somatic indel variant calling with Mutect2 6
Merging per-person VCF variant files 7
Preparing VCF file of TCGA gastric adenocarcinoma somatic variants for comparison 7
Annotating VCF variant files 7
Pre-analysis of variants and assigning variant status 7
Forced calling of variants based on evidence in sister biopsies 7
Filtering variants in VCF variant files 8
Assessing variant filters 10
Removing biopsies judged to be unreliable outliers 10
SNV/Indel Clonality assignment 10
Analysis of coverage data 10
Checking biopsy pairing 10
Mutation rate analysis and comparison to TCGA 11
Purity, copy-number, and aneuploidy analysis 11
Purity and ploidy analysis and calling CNV 11
Manual curation of PureCN purity/ploidy solution 11
Rerunning PureCN.R on curated data 12
Mutation burden calculation 12
CNV segment p-values 12
CNV gene p-values 13
Chromosome copy-number 13
Ancestral CNV events and TestCNVedgesOfSamplesOfPerson.R 13
Adjusting copy ratios, copy-numbers, and variant allele frequencies for purity and ploidy 13
Classifying into molecular subtypes 14

H. pylori infection assessment 15
Mutation druggability gene list construction 15
Mutation signature analysis 15
Phylogenetic analysis 16
Somatic SNV/indel-based trees 16
CNV-based trees 17
Event matrix weighting 19
Supplementary figures 19
Supplementary figure 1: Genes with clonal SNV mutations in at least one gastric cancer MSS or MSI patient. 20
Supplementary figure 2: Phylogenetic trees depicting inferred ancestral relationships of biopsies. 21
Supplementary figure 3: Phylogenetic trees for mutations in Figure 1. 22
Supplementary figure 4: Genes with clonal CNV mutations in at least one gastric cancer MSS or MSI patient. 23
Supplementary figure 5: Phylogenetic trees for mutations in Supplementary figure 1. 24
Supplementary figure 6: Phylogenetic trees for mutations in Supplementary figure 4. 25
Supplementary figure 7: Genes with only non-clonal mutations in all gastric cancer MSS and MSI patients. 26
Supplementary figure 8: Phylogenetic trees for mutations in Supplementary figure 7. 27
Supplementary figure 9: Mutation signatures found in clonal and nonclonal mutations in MSS and MSI samples. 28
Supplementary tables 29
Supplementary table 1. DNA repair genes that were somatic pan-cancer panel gene candidates. 29
Supplementary table 2. Genes whose CDS is less than 90% covered by the somatic pan-cancer panel. 30
Supplementary table 3. Molecular subtype patient count comparison with TCGA. 31
Supplementary table 4: Mutation signatures by molecular subtype, histology, and clonality in MSEQ vs. TCGA. 32
References 33

Supplementary figures and files referred to in the manuscript

The manuscript refers to **Supplementary figures 1-9** and **Supplementary tables 1-4**. These figures and tables can be found below in the sections titled "Supplementary figures" and "Supplementary tables". Data files as spreadsheets are referenced by the main manuscript and in this document by their filename ("Supplementary data file XX) and are available along with the manuscript.

Somatic pan-cancer panel design

The cancer panel gene composition is shown in **SuppDataFile1_PanCancerPanelGeneComposition.xlsx**. To design the panel, cancer gene lists obtained from the literature(1–39), Color Genomics test suite(46), Rahman cancer gene list(47), COSMIC Cancer Gene Census(40,41), genes mutated in one or more MSS samples of the TCGA stomach adenocarcinoma study(13,42,43), mutated in three or more of 36 in-house gastric cancer tumor samples, and a list of genes involved in DNA repair (**Supplementary table 1**) were collected into a master gene list with standardized HGNC(48) approved gene name(49), or, if the gene was not found in the HGNC list, with the NCBI Entrez(50) preferred gene symbol(51).

The *TERT* promoter at GRCh38 genomic coordinates chr5:1295163-1296162 was added.

Three sequences used for microsatellite instability testing were added (*MSI_D2S123* at chr2:51061283-51061523; *MSI_D5S346* at chr5:112877820-112878183; *MSI_D17S250* at chr17:38995794-38996034). Also, two more MSI sequences, *MSI_BAT25* and *MSI_BAT26*, were included by virtue of their overlap with genes *KIT* and *MSH2*.

Four sequences from the Epstein-Barr virus genome(57,58) and Fourteen sequences from the genomes of nine *H. pylori* strains were added for strain ELS37(59), strain 35A(60), strain 26695(61), strain B38(62), strain B8(63), strain 52(64), strain 2017(65), strain HUP-B14(66), and strain SouthAfrica20(67).

The final panel probes were designed using Agilent SureSelect XT2 Custom Capture technology(68) with a target region of 3.75 Mbp.

Anonymous Patient and Sample IDs

We assigned each patient and biopsy were an anonymous ID (I_#####) and S_#####, respectively. Each biopsy was also assigned a biopsy identifier in the form of N1, T1, T2, T3, etc., where "N" designates normal tissue and "T" designates tumor tissue.

DNA extraction and sequencing

We collected 2 to 5 individual tumor biopsies of gastric cancer tumors from 37 patients along with normal tissue biopsies adjacent to the tumors, for a total of 168 individual tumor biopsies and 37 normal biopsies. All biopsies were fresh-frozen tissue. DNA was captured with the somatic pan-cancer panel described above.

Each panel capturing run used 16 biopsy samples which were barcoded and pooled into a hybridization group. The goal was to sequence individual tumor biopsies at 1.5x more depth than normal biopsies.

The samples were sequenced on a total of 7 lanes in 3 batches on Illumina HiSeq 4000(69), using paired-end 150 base-pair sequencing. The hybridization groups, sequencing dates, and lane numbers were recorded for each sequenced sample aliquot in a tabbed table file named **PROJECT_info.txt** used within the computation pipeline, so these numbers were available for use by the pipeline (for example, for plotting sequencing depth of each sequenced sample aliquot).

There were 287 targeted genes that were covered less than 100%, and 24 of these were covered less than 90% (**Supplementary table 2**).

R packages used

The R programming language(71) was used for many aspects of the work described below, and within the R programs many R packages were used repeatedly:

1. readbitmap(72)
2. openxlsx(73)
3. cluster(74)
4. vcfR(75)
5. Rsamtools(76)
6. futile.logger(77)
7. VariantAnnotation(78)
8. GenomicRanges(79)
9. GenomicFeatures(79)
10. BSgenome(80)

11. TxDb.Hsapiens.UCSC.hg38.knownGene(81)

Reference genome and reference BED file construction

The *Homo sapiens* assembly 38 (hg38)(82) version of the human genome that was obtained from the GATK bundle database(83) was used as a starting point for constructing a reference genome. The following sequences were appended to the reference sequence: Epstein-Barr virus sequence (gi_94734074(58)) and these nine *H. pylori* variant genomes [NC_017063 (59), NC_017360 (60), NC_000915 (61), NC_012973 (62), NC_014256 (63), NC_017354 (64), NC_017374 (65), NC_017733 (66), NC_022130 (67)]

GENCODE(84) GFF3 version hg38 from was used to create a merged and ±12bp slop BED file of all CDS regions. The additional H. pylori and EBV regions were added to the BED file. Gene names were standardized to the HUGO preferred name(86).

Project BED file construction

Based on the design of the Agilent SureSelect XT2 Custom Capture we were provided with target region BED file 3025671_Covered.bed for the somatic pan-cancer. The panel design was lifted over to hg38 genome coordinates using the *liftOver* program(87) and padded by 200bp to create a padded target panel BED file. A target CDS BED file was also created by intersecting the CDS BED file above with the padded target panel BED file.

Shorthand names are used in the rest of this document when describing methods that made use of BED files:

- BED_TARGET_RGNS: the BED file of panel target regions including H. pylori and Epstein-Barr virus regions
- BED_MERGED_TGTS: version of BED_TARGET_REGIONS with overlapping regions merged
- BED_PADDED_TGTS: version of BED_MERGED_TGTS with 200 bp padding added
- BED_HPY: BED file of H. pylori regions
- BED_EBV: BED file of Epstein-Barr virus regions
- BED_PAD_REGIONS: BED file of the actual pad regions adjacent to target regions
- BED_NON_TARGET: BED file of genomic regions not in BED_PADDED_ TGTS
- RN_NAMED_TARGET_RGNS: BED that replaces BED_PADDED_TGTS column 4 gene names with R1, R2, …RN.

Read filtering and mapping

All FASTQ files (pre and post-processed) were QC'd using FASTQC(90) and FASTX(91).

FASTQ files were trimmed using Scythe(88) version 0.991 and Sickle(89) version 1.33. Scythe utilized options -q sanger -p 0.05 and illumina_adapters.fa file. Sickle was run with options pe -t sanger -g -q 30 -l 75, to produce reads with a minimum length of 75 bp and with an average PHRED Q score of 30 for the sequence in a sliding window.

A total of 15 biopsies from 3 persons were removed (9 tumor biopsies and 1 normal biopsy had poor coverage):

- I_8275_S_26900 through S_26905 (N1, T1-T5)
- I_9295_S26909 through S_26913 (N1, T1-T4)
- I_9708_S_26914 through S_26917 (N1, T1-T3)

We followed GATK(70) Best Practices for Variant Calling v2017. Reads were aligned to the reference using BWA-MEM(92) with options -L 0,0 -M -t 4 and sorted-merged using samtools(93) program suite version 1.8. Different BAM ReadGroups from the same biopsy were merged. Duplicate reads were marked using the Picard Tools(94) MarkDuplicates command of version 2.14.0. Indel realignment and base quality score recalibration was performed using GATK(95–97)version 3.7.

Quality control testing for artifacts, cross-sample contamination, and gender detection

Chromosome and target region mapped read counts were assessed using samtools view command with options -q 30 -f 2 -F 3844 (minimum mapping score of 30, reads must be properly aligned, must not be secondary or supplementary alignments, must pass platform/vendor quality controls, and must not be PCR optical duplicates) and bamtobed the bedtools program suite(98) version 2-2.27. Bar plots of the counts were produced. Target region counts were intersected target BED using the intersect command of the bedtools with the -wo argument. The number of base pairs in the regions of each of the output BED files was summed and aggregated across biopsies and plotted.

Hybrid selection and coverage metrics were generated BAM file using the CollectHsMetrics command of the Picard Tools program suite with options MINIMUM_BASE_QUALITY=22, MINIMUM_MAPPING_QUALITY=30,

PER_TARGET_COVERAGE, CLIP_OVERLAPPING_READS=false and interval file produced from the BED_TARGET_RGNS BED file**.** Coverage metrics were plotted and used as the first of two steps to confirm poor coverage of biopsies first identified with the FASTQC summary plots.

Mapping quality metrics were assessed using CollectMultipleMetrics command of the Picard Tools. Additional mapping quality metrics were generated using bamqc version 2.2 of the Qualimap(99) program with using BED_TARGET_RGNS BED file, to produce a per-biopsy directory of metrics files.

Basepair-level coverage was generated using samtools view with options -q 30 -F 3840 and bedtools coverage command with options -nonamecheck -sorted -d and the panel target regions. These files were aggregated across all biopsies and plots were used as the second of two steps to confirm poor coverage of biopsies prior to removing them from the analysis.

Sequencing artifact statistics were collected using CollectSequencingArtifactMetrics command of the GATK program suite version 4.0.6.0 with options --INCLUDE_UNPAIRED=true --MINIMUM_INSERT_SIZE 10 --MINIMUM_MAPPING_QUALITY=30 --MINIMUM_QUALITY_SCORE=22. Results were aggregated across biopsies and plotted. That data shows that the mean sequencing error rate is about 0.03%, with a peak rate of 0.07% for C>T transitions, which was deemed highly favorable.

Cross-sample contamination testing was performed using GATK program suite version 4.0.6.0 GetPileupSummaries, which utilized a hg38 liftedOver version of the Gnomad version r2.0.1 human exome SNP dataset(100), and CalculateContamination. These were aggregated and plotted. The data shows that cross-sample contamination was less than 1.5% for all biopsies except P26.3 (5%) and P17.3 (2.5%). These contamination levels were deemed acceptable.

Sequence data for each biopsy was tested to verify that gender matched the recorded gender of the person; for each biopsy sample, the ratio of number of reads mapping to chromosome Y to total number of reads was calculated. If the ratio exceeds 0.00025, the biopsy is deemed male, else female. All biopsies were found to match the expected gender.

After quality control, the number of passing biopsies was reduced to 115 individual tumor biopsies from 32 patients.

<u>Extraction of unaligned and Epstein-Barr and H. pylori reads</u>

Amount of unaligned reads was assessed using samtools view command with argument -F 2. The number of unaligned reads were plotted.

Reads mapping to Epstein-Barr virus or *H. pylori* bacterial genomes were extracted using samtools view with option -F 3844. The number of reads were aggregated and plotted.

<u>MSI assessment</u>

Microsatellite instability was assessed using MSIsensor(101) scan and msi. The msi command used options -f 0.05 -c 20 -l 5 -p 10 -m 50 -q 3 -s 5 -w 40 -u 500 -b 1 -x 0 -y 0 and paired tumor-normal BAM. The results were aggregated and plotted.

<u>Variant calling</u>

Panel of Normals construction

A *panel of normal (PON)* was used with GATK Mutect2(102) caller using the Best Practices procedure outlined by the BROAD Institute(103). Gnomad v2.0.1 hg38 VCF was used for germline SNPs.

SNV variant calling with multiSNV

SNV variants were called using multiSNV(104), which calls germline and somatic SNVs jointly (simultaneously) in all biopsies of a person.

The multiSNV program was modified in several ways:

1. Modified to improve calling of germline SNVs. The original code called a variant when the major alleles in normal and tumor were different, but this missed germline calls when 100% of reads in both normal and tumor were alternate allele reads (and therefore were the major allele). The code was modified to change this condition to call a variant if the major allele differed from the reference allele.
2. Modified to count reads with lower mapping quality that the normal quality threshold. Option -x (--minMapQualX) was added, taking a mapping quality number as an argument. This causes counts of reads with

mapping quality >= the specified mapping quality to be accumulated and written to the output VCF file in the form of XD and XCOUNT FORMAT keys, with XD having counts of reference and alternate allele reads at or above that mapping quality (like AD) and XCOUNT having counts of reads with bases A,C,G,T at or above that quality (like BCOUNT).

3. Modified to speed up the program. The program was modified to keep the reference FASTA file open while processing, and cache reference information for faster access.

SNV variants were called using the multiSNV program (version v2.3-15 with above modifications added) with options --include-germline 1 --include-LOH 1 --minBase 22 --minMapQual 30 --minMapQualX 1 --dmin 15 --udmin 3 --dmax 500 --medianN 40 --medianT 40 --mva 1 --low_depth 15 --weak_evidence 0.01 --normal_contamination 0.03 --minVariantReadsForTriallelicSite 2 --flag-homopolymer 5 --mu 0.0001 --Rmdups 1 --add_UD 1 --conv 0 --bed <BED_PADDED_TGTS> -N <NUM_BIOPSIES>. The passing variants were selected using GATK SelectVariants.

Germline SNVs were extracted from the per-person multiSNV VCF using bcftools view command to extract non-wildtype variants for the normal, requiring minimum depth of 15 and minimum VAF of 0.2. Variants were flagged with INFO flag GNOMAD if they appeared in the <GNOMAD_EXOME_VCF> using the bcftools annotate command.

Tandem Repeats Finder, RepeatMasker, and WindowMasker tracks(105) were downloaded from the UCSC Table Browser version hg38 (106–109), The INFO flags PON, QUES, and RPTS were added to the per-person germline VCF files using BED files to specify the applicable regions: the PON GATK, PON questionable regions, and simple repeats BED files. Germline variants were filtered to produce a VCF of likely germline mutations that were not common population SNPs or in questionable call areas or areas containing simple repeats.

Somatic indel variant calling with Mutect2

Somatic indel variants were called using GATK Mutect2(102) version 4.0.6.0, with options which calls somatic SNVs and indels from paired normal and tumor input BAM files. The SNV calls were filtered out (multiSNV SNV calls were used instead) and only the indel calls were used. Mutect2 was run with options --create-output-bam-index --create-output-variant-index --af-of-alleles-not-in-resource 0.0000025 --disable-read-filter MateOnSameContigOrNoMappedMateReadFilter --min-base-quality-score 22 --seconds-between-progress-updates 30 --panel-of-normals PanelOfNormals.gatk.vcf.gz --germline-resource <GNOMAD_EXOME_VCF> to produce per-tumor VCF files of somatic variants and per-tumor BAM files of realigned reads.

The Mutect2 output VCF files were filtered using the FilterMutectCalls command of the GATK program suite with options --annotate-with-num-discovered-alleles --max-alt-allele-count 1 --max-strand-artifact-probability 0.9 --min-median-base-quality 22 --min-median-mapping-quality 30 --unique-alt-read-count 5 --seconds-between-progress-updates 30 --smith-waterman FASTEST_AVAILABLE -OVI --contamination-table <TUMOR CONTAMINATION FILE> to produce per-biopsy confident somatic variant call set VCF files.

Sequencing artifacts were removed from these VCF files using the GATK FilterByOrientationBias with options **--**artifact-modes G/T --artifact-modes C/T -OVI -P to produce per-biopsy artifacts-removed somatic variant call set VCF files. The non-passing variants were filtered using GATK SelectVariants.

Merging per-biopsy VCFs

This project uses multi-regional sequencing with multiple tumors per biopsy, yet the Mutect2 output is on a per-biopsy basis. The Mutect2 per-biopsy somatic indel VCF files of each person were merged to form per-person VCF files of somatic indels. This was a long multi-step procedure, established so as to ensure that the final merged file contained only those somatic variants for which there was solid evidence of either mutant or wild type genotype from either Mutect2 or the GATK caller HaplotypeCaller from each biopsy of the person. The procedure was:

1. Generate a union set of passing germline and somatic variants for each person
2. GATK HaplotypeCaller version 4.0.6.0 was used to call variants that exist in the union set but were not called in the biopsy
3. All per-person biopsy VCFs were then merged
4. The bcftools isec command with options **-**n=<NUM_TUMORS> -c all -w 1 to intersect all per-biopsy merged VCF files of variants present in all biopsies
5. The bcftools isec command with options -n=2 -c all -w 1 calls at all loci where a mutation occurred in ANY of that person's tumors.
6. The bcftools isec command with options **-**n=2 -c all -w 1, to produce normal VCF files containing calls at all loci where a mutation occurred in ANY of that person's tumors.
7. The final VCF was created by merging the per-person normal all-loci VCF file and all of the person's per-biopsy enhanced all-loci VCF files.

Merging per-person VCF variant files

Per-person MultiSNV VCF files were merged using the bcftools merge to produce a single project VCF file containing all MultiSNV germline and somatic SNV variants called in any biopsy of any person. In the same way, all of the per-person Mutect2 merged VCF files were merged to produce a single project VCF file containing all Mutect2 indel somatic variants called in any biopsy of any person.

Preparing VCF file of TCGA gastric adenocarcinoma somatic variants for comparison

Utilizing cBioPortal(42), datasets for Nature 2014 (PubmedID: 25079317) downloaded on 11/2016 - data_mutations_extended.txt and data_clinical.txt were utilized to generate a VCF file in GRCh38 using a custom Rscript. The VCF file was then filtered according to padded targets region of the panel.

Annotating VCF variant files

The two merged project VCF files from multiSNV and mutect2 variant calling, and the TCGA somatic variant VCF file, were annotated Annovar(110) with the following datasets: refGene,1000g2015aug_all,avsnp150,cosmic86,clinvar_20170905,gnomAD_genome_ALL,ExAC_ALL, and hrcr1. Additionally, the VCF files were annotated with four INFO flag tags (PON, STR, RPT, WMR) (see above). The VCF files are primary outputs of the project, and are named AllVars.multiSNV.anno.marked.vcf.gz, AllVars.mutect2.anno.marked.vcf.gz, and TCGA_GC_data_mutations_extended_hg38.panel.norm.anno.marked.vcf.gz.

Pre-analysis of variants and assigning variant status

Analysis of variant status was performed using a custom Rscript that read the two annotated and flag-marked VCF files for variants called by multiSNV and Mutect2. It calculated the following values, which are used in filtering below:

- GTval: variant genotype value, one of "WT" (wild type), "HET" (germline), "HOM" (germline), "BIA" (bi-allelic), "GRM" (germline indeterminate zygosity), "SOM" (somatic), "LOH" (somatic with loss of heterozygosity), "BOTH" (both germline and somatic mutations at same locus), created by analyzing the FORMAT field genotype strings (e.g. 0/0, 0/1, etc.).
- VarStat: variant status, one of "WT" (wild type), "GRM" (germline), "SOM" (somatic), "LOH" (somatic with loss of heterozygosity), "UNK" (unknown or unusual/rare), created from the SS FORMAT tag if available (multiSNV only), else from GTval (Mutect2 only):
- VarFunctional: examines the Annovar functional consequence annotations Func.refGene and ExonicFunc.refGene and assigns a single functional consequence keyword to each variant, one of synonymous, nonsynonymous, stoploss, stopgain, inframeindel (indel), frameshift (indel), unknownExonic, splicing, intronic, UTR (could be either UTR5 or UTR3), TERT_PROMOTER, ncRNA (non-coding RNA), intergenic (includes up/downstream), other

Forced calling of variants based on evidence in sister biopsies

Manual examination of variant call results revealed examples of cases where it appeared that multiSNV or Mutect2 had made an incorrect call, calling a genotype as wildtype in one or more tumors when it was called as a somatic variant in one or more other tumors. This is expected in Mutect2, which does not do joint-sample calling, and in multiSNV is due to its using a conservative estimate of joint probabilities for mutations that are clonal in nature. Based on our manual review of the data, we implemented a special *forced variant calling* algorithm using special R code. It read the multiSNV and Mutect2 merged VCF files (all patients, all biopsies in one VCF file) and applied an algorithm to test whether a given variant called in one biopsy should be called in another sister biopsy. If the algorithm indicated the variant should be called, a file of these forced variant calls was created, and subsequently whenever one of the VCF files was read, the forced variant call file was also read and used to modify the VCF file data to include the forced variant calls. The algorithm was as follows. For each variant of each sample in the VCF file in question, a wild-type call is changed to a somatic mutation call if:

1. The sample under consideration is a TUMOR sample
2. The sample locus under consideration is called wildtype
3. The paired NORMAL sample is called wildtype at that locus
4. The locus has only a single record in the VCF file
5. Read count, ref and alt depth, and purity/copy-number-adjusted VAF data (described later) is available for the sample at that locus
6. In the sample under consideration, the read count for the alt allele is >= 2 OR is equal to 1 and the copy-number-adjusted VAF for the allele is >= 0.01
7. There are one or more sister TUMOR samples for the same person
8. At least one sister TUMOR is called variant

9. All sister TUMORS that are called variant have their first alternate allele equal to the alt allele in the sample under consideration
10. At least one sister TUMOR that is called variant has its first alternate allele read count >= 6
11. That same sister TUMOR has PureCN gene CNV data available for the gene containing the variant, and its purity/copy-number-adjusted VAF for its first alternate allele is >= 0.025

Filtering variants in VCF variant files

The common R code used for variant analysis included the definition of filters and application of them to the variant list to create filtered subsets of variants. Each filter has a name which starts with "grm" for filters that select only germline variants and with "som" for those that select only somatic variants. For the purpose of describing somatic variants in the main part of the manuscript, the following filters were used:

- somPanelGenes: Selects somatic variants described in the manuscript as the full set after filtering out false positives and sex chromosome mutations. Referred to as the "final list" of variants.
- somCodSplNoSyn: Selects somatic variants described in the manuscript as non-silent coding or splicing mutations

For identifying germline mutations, the following filter was used:

- grmReliable: Selects germline variants that are reliably called.

For comparing mutation rate between TCGA and this project, the following filter was used:

- somCDS_CodSpl: Selects somatic variants in coding or splicing regions of panel CDS target regions. A similar filter was applied to the TCGA mutation data to select mutations for computing TCGA mutation rate, so that the comparison fairly compared mutations in the same regions.

Each filter consists of one or more filter actions. Each filter is actually THREE filters: (1) a variant filter that selects a subset of all variants; (2) a variant-person filter that selects, for each variant, one or more persons carrying that variant; (3) a variant-biopsy filter that selects, for each variant, one or more biopsies carrying that variant. If a variant is not selected in (1), it is also not selected for any person in (2) or any biopsy in (3). If a variant is not selected for some person in (2), it is also not selected for any biopsy of that person in (3). Some filter actions operate at the level of the variant (1), some at the level of person (2), and some at the level of biopsy (3), but all three filter types can be affected by each action. In the following filter descriptions, the filter name is shown after the bullet, and a numbered list of filter actions follows. *The descriptions are written in terms of which variants pass the filter and are accepted.*

Somatic variant filters are as follows:

- som: somatic variants in tumor biopsies only. Selects a set of 0 or more tumor biopsies for each variant.
  (1) select TUMOR biopsies only
  (2) select VarStat = SOM or LOH
- somReliable: reliably-called somatic variants in TUMOR biopsies only.
  (1) above som actions applied first
  (2) read depth in NORMAL >= 20
  (3) read depth in ALL TUMORS OF PERSON >= 30
  (4) alternate allele depth in AT LEAST ONE TUMOR >= 5
  (5) NORMAL alternate allele depth = 0 or =1 and VAF <= 0.01
  (6) the variant is called in more than one sample of the person OR it is called in only one sample and the other samples of that person have a total allele count for other than the ref and alt alleles of no more than 1 + 10% of the variant sample's alt allele count
  (7) if GQ tag is present (multiSNV only), NORMAL GQ <= 1e-8
  (8) if GQ tag is present (multiSNV only), GQ <= 1e-8 in all tumors of the person
  (9) STR tag not present (not a simple tandem repeat)
  (10) RPT tag not present (not a RepeatMasker repeat)
  (11) WMR tag not present (not a WindowMasker repeat)
  (12) Reject loci that seem to be experiencing sequencing artifacts causing higher than normal sequencing error rates, by examining NORMAL biopsies for loci that are called WT but have far fewer than the expected number of reference allele reads (given the total read depth and the known sequencing error rate of 0.0008), or that are called HET but have far fewer than the expected number of alternate reads (given the total read depth and an expected probability of an alternate allele read being no lower than 0.4). At any given locus, if two or more NORMAL biopsies show such unusual read counts, reject ALL VARIANTS IN ALL BIOPSIES at that locus. Tests are based on the binomial distribution, and the test failure thresholds are computed dynamically for each locus that is tested, in order to achieve a total over all variants of 5 or fewer false positive rejections with probability >= 0.95. The dynamic threshold is computed in two steps. First, a single probability pMin is computed, such that IF a locus is rejected at probability pMin under null hypothesis conditions, then there will be fewer than 5 false positive rejections with probability 0.95. Second, for each locus, a single probability threshold is computed for rejecting a NORMAL at that locus. For a given

NORMAL, its test fails (rejection) if the number of reads of the requisite type (reference or alternate) has a probability of occurrence under the null hypothesis of less than this threshold probability. The threshold probability is chosen such that the probability of rejection of the locus under the null hypothesis (when 2 or more NORMALS fail their test) is pMin (and since each locus has a varying number of NORMALS with genotype data, that probability changes depending on the locus).
   (13) variant is not present in the GnomAD genome ALL database or its population frequency is < 0.0001 in that database
   (14) variant is not present in ExAC database release 1 or its population frequency is < 0.0001 in that database
- somExcludeXY: select reliable somatic variants not found on chromosomes X or Y
   (1) above somReliable actions applied first
   (2) variant position is not in chrX or chrY
- somPanelGenes: select reliable somatic variants in LCC panel genes, not found on X or Y
   (1) above somExcludeXY actions applied first
   (2) variant lies in a region given by the BED_MERGED_TGTS bed file
   (3) there is at least one LCC panel gene within the variant's gene.RefGene list created by Annovar
- somCodSplNoSyn: select reliable non-silent coding/splicing somatic variants in LCC panel genes, not on X or Y
   (1) above somPanelGenes actions applied first
   (2) VarFunctional is one of nonsynonymous, stoploss, stopgain, frameshift, inframeindel, unknownExonic, splicing, TERT_PROMOTER, other
- somCDS_CodSpl: select reliable coding/splicing somatic variants in CDS target regions of LCC panel genes, not on X or Y
   (1) above somPanelGenes actions applied first
   (2) VarFunctional is nonsynonymous, synonymous, stoploss, stopgain, frameshift, inframeindel, unknownExonic, splicing, TERT_PROMOTER, other
   (3) variant lies in a region given by the BED_TARGET_RGNS bed file

Germline variant filters are as follows:
- grm: germline variants in normal biopsies only. Selects a set of 0 or more normal biopsies for each variant.
   (1) select NORMAL biopsies only
   (2) select VarStat = GRM
- grmReliable: reliably-called germline variants in NORMAL biopsies only.
   (1) above grm actions applied first
   (2) read depth in NORMAL >= 20
   (3) alternate allele depth in NORMAL >= 5
   (4) if GQ tag is present (multiSNV only), NORMAL GQ <= 1e-8
   (5) STR tag not present (not a simple tandem repeat)
   (6) RPT tag not present (not a RepeatMasker repeat)
   (7) WMR tag not present (not a WindowMasker repeat)
   (8) no NORMAL biopsies with a genotype other than 0/0 and read depth >= 30 may have VAF < 0.16. A variant is filtered out from ALL biopsies if ANY NORMAL BIOPSY fails this test. This would indicate a likely artifact problem at the locus. Sequencing errors will cause occasional NORMALS with very low VAF, but those should be called with a genotype of 0/0.
   (9) Reject loci that seem to be experiencing sequencing artifacts causing higher than normal sequencing error rates (same method as somReliable filter described above).

The following TCGA data variant filter was created for extracting somatic variants from TCGA data in the same regions as targeted by the LCC panel, for the purpose of comparing mutation rate between TCGA and this project. This filter matches as closely as possible the somCDS_CodSpl filter above, given the available mutation attributes.

- TCGA.somCDS_CodSpl: select somatic variants for comparison to MSEQ project variants to compare mutation rates:
   (1) select TUMOR biopsies only (TCGA data only contained tumor biopsy data)
   (2) VarStat = SOM or LOH
   (3) read depth in ALL TUMORS OF PERSON >= 30
   (4) alternate allele depth in AT LEAST ONE TUMOR >= 5
   (5) STR tag not present (not a simple tandem repeat)
   (6) RPT tag not present (not a RepeatMasker repeat)
   (7) WMR tag not present (not a WindowMasker repeat)
   (8) total number of somatic mutations < 400 (else presumed to be WGS rather than WES sample; samples weren't annotated with type of sequencing in the downloaded TCGA file). The number 400 was chosen after

examining all samples for number of mutations and observing a bimodal distribution that was attributed to type of sequencing.

(9) variant is not present in the GnomAD genome ALL database or its population frequency is < 0.0001 in that database

(10) variant is not present in ExAC database release 1 or its population frequency is < 0.0001 in that database

(11) variant position is not in chrX or chrY

(12) variant lies in a region given by the BED_MERGED_TGTS bed file.

(13) there is at least one LCC panel gene within the variant's gene list created by Annovar

(14) VarFunctional is nonsynonymous, synonymous, stoploss, stopgain, frameshift, inframeindel, unknownExonic, splicing, TERT_PROMOTER, other

(15) CDStgtRgns: variant lies in a region given by the BED_TARGET_RGNS BED file

Assessing variant filters

For each of the variant filters listed above, a bar plot was made with one bar per sample, and each bar being a stack of bar segments, one segment for each filtering action within that filter, and with each segment's height corresponding to the number of variants removed by that action for that sample. These plots were reviewed to assess how each filtering action in each filter was behaving, to look for any unexpected or problematic filtering actions.

Removing biopsies judged to be unreliable outliers

A bar plot was made, with one bar per biopsy and with each bar being two stacked bar segments, one segment showing number of "reliable" somatic variants in the biopsy (passing filter "somReliable") and the other showing the number of "unreliable" somatic variants (passing filter "som" but not passing "somReliable"). Review of this plot showed three biopsies that were judged to be unreliable outliers, and they were subsequently removed from the analysis:

1. I_6579_S_26840 (T1): this biopsy had over 4000 total somatic variants, more than 10 times its sibling biopsies.
2. I_9709_S_26846 (T3): this biopsy had almost twice as many somatic variants (225) as its sibling biopsies, all of which had a fairly uniform number (around 125) of somatic variants.
3. I_19343_ S_26861 (T4): this biopsy had more than twice as many somatic variants (260) as its sibling biopsies, all of which had a fairly uniform number (around 100) of somatic variants.

SNV/Indel Clonality assignment

For each SNV or indel variant in each person, clonality is assigned from the GTval values as follows:

- CLONAL: NORMAL is not germline variant, TUMORS are all somatic variant (for that variant).
- SUBCLONAL: NORMAL is not a germline variant, more than one but not all TUMORS are somatic variant.
- PRIVATE: NORMAL is not germline, only one TUMOR is somatic variant.
- GERMLINE: NORMAL and all TUMORS are germline variant.
- GONE: the variant, whatever sample it was called in, has been filtered out by the grmReliable or somPanelGenes filter.
- NONE: no variant was called in the NORMAL or any TUMORS.

Analysis of coverage data

A custom R program was used to compute and plot numerous coverage statistics (here, "minimum depth" means depth of at least 15X):

- mean mapped coverage of each biopsy across all target regions
- a series of global coverage statistics
- for each of the following, the mean target region depth (across target regions and/or biopsies), standard deviation of depth, and percent of target regions and/or biopsies with minimum depth:
    a) each biopsy
    b) each individual
    c) each target region specified in the RN_NAMED_TARGET_RGNS BED file
    d) each targeted gene
    e) each DNA pool hybridization group

Checking biopsy pairing

All pairs of biopsies (regardless of owning individual) were compared for similarity by computing the cosine similarity of the variant allele frequencies (VAFs) of all germline variants present in one or both biopsies and having a depth in both biopsies lying between 80% and 250% of the mean depth of all the biopsy's variants. A cosine similarity threshold is chosen by

seeking a minimum in the empirical distribution of the cosine similarities from all pairs (since most pairs are mismatch, but a number of pairs will batch, giving a bimodal distribution). As a result of this testing, two mismatches were discovered, leading to 6 biopsies being removed from the analysis:

- I_26180_S_26858 (T1): This tumor biopsy did not match its normal biopsy
- I_9298_S_26895 through S_26899 (N1, T1-T4): The normal biopsy matched the I_19343_S_26857 (N1) biopsy and mismatched its tumor biopsies.

Mutation rate analysis and comparison to TCGA

Somatic mutations were filtered using the somCDS_CodSpl.samp filter on a per-sample basis. TCGA somatic mutations from the preprocessed TCGA GC VCF file were filtered using the TCGA.somCDS_CodSpl.samp filter on a per-sample basis. The mutation rates of each MSEQ sample, the mean rate of the MSEQ MSI and non-MSI samples, and the mean rate of the TCGA MSI and non-MSI samples were computed, along with standard error of the mean for each mean rate, and these were plotted on a bar plot for comparison of mutation rates.

Purity, copy-number, and aneuploidy analysis

Copy-number analysis, including tumor purity and ploidy analysis, was performed using PureCN(117), version 1.16.0, that was designed for use with targeted-panel data. A genome mappability file was created using the GEM library(118) program version 1.778 beta and the GATK bundle genome reference file Homo_sapiens_assembly38.fasta. The PureCN IntervalFile.R program was run to create text and BED intervals files, using options --infile <FILTERED_BED_FILE> --fasta Homo_sapiens_assembly38_ LCCpanel.fasta --genome hg38 –force Homo_sapiens_assembly38_LCCpanel.gemV2_100.mappability.bigwig --offtarget --offtargetwidth=200000. The PureCN Coverage.R program was run on each biopsy's final recalibrated BAM files (both NORMAL and TUMOR) with the interval file and options --seed 123 --force --removemapq0, producing four PureCN coverage files per biopsy.

Per-tumor-biopsy VCF files of variants were prepared for PureCN by merging, on a per-tumor-biopsy basis, the two per-person multiSNV and Mutect2 pass-filtered normalized VCF files for each biopsy.

Purity and ploidy analysis and calling CNV

The PureCN PureCN.R program was run on each tumor biopsy's PureCN coverage file and PureCN VCF file, using options --normaldb <normalDB .rds file> --normal_panel <mapping bias .rds file> --intervals <PureCN intervals file> --snpblacklist < simple repeats BED file> --genome hg38 --seed 123 --postoptimize --force --error=0.001 --padding=100 --alpha=0.005 --minpurity=0.1, to call CNV segments in the biopsies.

Manual curation of PureCN purity/ploidy solution

A custom R program named ManualCuratePureCNsample.R was run for each tumor biopsy to analyze the PureCN run's results and determine whether or not the chosen purity/ploidy solution should be altered and the resulting manual curation of purity/ploidy established as the new chosen solution. The program was run with options --minLikelihoodFrac 0.7 --minPeakHeatFrac 0.7 --minProductFrac 0.7 --minTgtPloidy 1 --maxTgtPloidy 6 --minTgtPurity 0.1 --maxTgtPurity 1.0 --maxNonHighAdjVAF 1.2 --minNonLowAdjVAF -0.2 --maxVarsBadVAF 2 --maxFracVarsBadVAF 0.2 --CR1_thresh 0.15 --roundCRstart 0.01 --minCRsegs 5 --minCRsegLen 10e6 --roundCRdelta 0.01. It uses three methods to detect poor PureCN solutions. The first method is based on the observation that sometimes PureCN's maximum likelihood solution has a heatmap value significantly lower than the peak heatmap value, and in those cases the solution often appears to be wrong. The method is to reject as unacceptable those PureCN solutions that have ANY of the following conditions:

1. PureCN total likelihood value ranked within bottom --minLikelihoodFrac total likelihoods among all solutions.
2. PureCN heatmap value ranked within bottom --minPeakHeatFrac heatmap values among all solutions, but if this rejects all solutions, instead choose the one solution (after the previous step) that has the highest heatmap value.
3. A combination of the above two factors, obtained by taking the product of the PureCN total likelihood and heatmap values, and rejecting those solutions with a product ranked within the bottom –minProductFrac products among all solutions. If this rejects all solutions, instead choose the one solution (after the previous steps) that has the highest product value.

The second method is based on the values of the variant allele frequencies after adjusting them for purity, ploidy, and copy-number. It is observed that sometimes, a large fraction of these VAF values are greater than 1 or less than 0, suggesting that the PureCN purity/ploidy solution is incorrect and caused the adjusted VAF to be too high or low. The method is to reject as unacceptable those PureCN solutions that have BOTH of the following conditions:

1. Number of PureCN-filtered variants with purity/ploidy/copy-number-adjusted VAF (variant allele frequency) outside the acceptable range (which is minNonLowAdjVAF to maxNonHighAdjVAF) is > maxVarsBadVAF
2. Fraction of such variants is > maxFracVarsHighVAF

After applying these two algorithms, additional steps are applied to eliminate more solutions:

1. If there is more than one remaining non-rejected solution, reject those with ploidy outside the range minTgtPloidy..maxTgtPloidy, unless this rejects all solutions.
2. If there is more than one remaining non-rejected solution, reject those with purity outside the range minTgtPurity..maxTgtPurity, unless this rejects all solutions.
3. If there is more than one remaining non-rejected solution, choose the one with the highest total log likelihood.

Another function of ManualCuratePureCNsample.R is to read the PureCN segments output file and estimate a biopsy copy ratio that corresponds to the presumed no-CNVs copy ratio. Typically, there will be long segments on many separate chromosomes where the PureCN copy ratio is approximately but not exactly 1, typically differing by no more than 0.01 between such segments. A Wilcoxon test comparing the copy ratios of the markers within such segments to the expected no-CNVs copy ratio of 1.0 often produces a very small p-value because the copy ratios of the markers are slightly different than 1.0 and exhibit almost no variance. This suggests that PureCN does not normalize the copy ratio data as well as it might, as even in a highly disturbed cancer genome there should be a significant genome fraction in dosage balance and having the same copy ratio. That copy ratio should be identified as the no-CNVs copy ratio for the purpose of performing a Wilcoxon test for amplification or deletion. ManualCuratePureCNsample.R uses an algorithm to identify segments whose unadjusted raw copy ratio is very close to 1, and where the number of such segments is relatively high compared to segments with other copy ratios:

1. Eliminate from consideration all segments whose copy ratios are not in the range 1 ± CR1_thresh. If this results in eliminating ALL segments, increase CR1_thresh by 10% and try again, repeating until at least minCRsegs of at least minCRsegLen total length are obtained.
2. Beginning with roundCR = roundCRstart, round each of the remaining segments' unadjusted copy ratios to the nearest multiple of roundCR.
3. Find the most common rounded copy ratio. If more than one copy ratio occurs with the same maximum occurrence frequency, choose the ratio closest to 1.
4. If the number of segments in that most-common-copy-ratio is < minCRsegs, or if the total length of those segments is < minCRsegLen, increase roundCR by roundCRdelta and repeat the above steps, until an acceptable most-common-copy-ratio is found.

The mean copy ratio of those segments having a rounded copy ratio equal to the most-common-copy-ratio is computed and added as a comment to the new curation .csv file, in the 'Comment' column, as for example the comment: 'LOW PURITY;EXCESSIVE LOH;CR1=1.0233'. This number can be divided into any raw copy ratio from PureCN to move it in the direction of what is surmised to be a more accurate copy ratio, and resulting in those most-common-copy-ratio segments having an adjusted copy ratio that is equal to or at least closer to 1.0. The program writes a new copy of the .csv file containing PureCN purity and ploidy solution and the computed CR1 value in a new subdirectory named “curated” (done on a per-tumor-biopsy basis).

Rerunning PureCN.R on curated data

For each tumor biopsy for which a different purity/ploidy solution was chosen in the preceding step, the PureCN PureCN.R program was run a second time, with the --rds option specified to request that it recompute output files using the new purity/ploidy solution.

Mutation burden calculation

The PureCN Dx.R program was run on each tumor biopsy’s PureCN .rds file, with options --callable <BED_TARGET_RGNS> --exclude hg38_simpleRepeats_merged.bed –force, to estimate the mutation rate. Per-tumor-biopsy comma-separated variable output files are created.

CNV segment p-values

A custom R program named CreateCNVsegmentPvals.R was run for each tumor biopsy, with options --fdr 0.01 --CR_thresh 0.1 --CRdif_thresh 0.25 --CRdif_frac 0.25 and input files BED_TARGET_RGNS, PureCN curation, .rds data, and segment copy-number files, which assigned each segment a p-value by using a Wilcoxon test:

1. For each segment, get the PureCN markers overlapping it from the PureCN .rds file and adjust their copy ratios by dividing them by the presumed no-CNVs copy ratio (CR1 value obtained from the curation file comment).
2. Further adjust the marker copy ratios for purity and ploidy (see below), using that of the PureCN solution given in the PureCN curation file. Those copy ratios that were originally equal to the CR1 value are now exactly 1.
3. Use a single-tailed Wilcoxon single-sample test to test the marker adjusted copy ratios to see if they satisfy the null hypothesis of having a population mean rank of 1 (or for the alternative hypothesis, that it is less than or more than 1). Two tests are done, one for less than, one for more than. Select the smaller of the resulting p-values as the result p-value for the gene. If the first p-value is selected, the gene is annotated as having a copy ratio < 1 (DELetion), and for the second p-value, > 1 (DUPlication).

A multiple-testing-adjusted q-value was associated with each such p-value using the Benjamini and Hochberg (FDR) method. Each gene is annotated with adjusted copy ratio, mean marker adjusted copy ratio p-value, and q-value.

CNV gene p-values

A custom R program named CreateCNVgenePvals.R was run for each tumor biopsy, with option --ttest and as input, PureCN curation, .rds data, and gene copy-number files, which assigned each gene a p-value by using a Student's t-test:

1. For each gene, get the PureCN markers overlapping it from the PureCN .rds file and adjust their copy ratios by dividing them by the presumed no-CNVs copy ratio (CR1 value obtained from the curation file comment).
2. Further adjust the marker copy ratios for purity and ploidy (see below), using that of the PureCN solution given in the PureCN curation file. Those copy ratios that were originally equal to the CR1 value are now exactly 1.
3. Use a single-sample Student's t-test to test the marker adjusted copy ratios to see if they satisfy the null hypothesis of having a normal population mean equal to 1 (or for the alternative hypothesis, that it is less than or more than 1). Two tests are done, one for less than, one for more than. Select the smaller of the resulting p-values as the result p-value for the segment. If the first p-value is selected, the segment is annotated as having a copy ratio < 1 (DELetion), and for the second p-value, > 1 (DUPlication).

A multiple-testing-adjusted q-value was associated with each such p-value using the Benjamini and Hochberg (FDR) method. Each segment is annotated with adjusted copy ratio, mean marker adjusted copy ratio p-value, q-value, and names of specified target genes contained within it.

Chromosome copy-number

A custom R program named GetChrCNV.R was run for each tumor biopsy, with options --ploidyTolForGain 0.6 --ploidyTolForLoss 0.6 --fracForAltered 0.66, to analyze the PureCN copy-numbers for CNV segments along the genome, in order to estimate copy-numbers and loss-of-heterozygosity for whole chromosomes and chromosome arms. The program used centromere positions given by downloading the human genome version GRCh38 centromeres track data using the UCSC Table Browser(106–109) on 21-May-2018, then extracting columns 2-4, sorting in GRCh38 chromosome order with sort command in bedtools suite, and merging overlapping regions with the merge command of the bedtools suite. The GetChrCNV.R program read the PureCN curation file of sample purities and ploidies and the PureCN segments file with mean marker copy-number of each CNV segment (adjusted for purity/ploidy by CreateCNVsegmentPvals.R). It computed several values for each chromosome and chromosome arm:

1. PureCN purity/ploidy estimates and number of PureCN CNV segments in the chromosome or arm
2. mean adjusted copy ratio and copy-number of the segments.
3. rounded marker adjusted copy-number that covers the most CNV segment distance for chromosome or arm segments with that copy-number
4. fraction of distance covered by the CNV segments that have that most frequent rounded adjusted copy-number
5. fraction of chromosome marker distance over which the segments' adjusted copy-number was > ploidy+0.75
6. fraction of chromosome marker distance over which the segments' adjusted copy-number was < ploidy-0.75
7. fraction of chromosome marker distance over which the PureCN's estimated segment minor copy number was 0 and not flagged by PureCN as unreliable and where total rounded copy number lies between 1 and 6 (loss-of-heterozygosity, LOH)
8. flags for fractions in 4-7 above exceeding the threshold of 0.66 of the chromosome marker distance
9. number of segments available to estimate copy ratio
10. number of segments with gain if the gain flag is set, or number with loss if the loss flag is set
11. mean copy ratio/number of those segments with gain if gain flag set, or with loss if loss flag set

Ancestral CNV events and TestCNVedgesOfSamplesOfPerson.R

The CNV analysis performed by PureCN is done individually per biopsy to try to identify ancestral CNV events that are common to more than one biopsy of an individual. PureCN does no per-person analysis, so a custom R program was developed for this purpose, named TestCNVedgesOfSamplesOfPerson.R. The fundamental idea behind the program is that ancestral CNV events should have CNV segments with the same start and/or end position within the biopsies descending from the ancestral cell.

Adjusting copy ratios, copy-numbers, and variant allele frequencies for purity and ploidy

The raw output from PureCN programs normally includes raw, unadjusted copy-numbers (for variants, segments, and genes). The purity and ploidy of a biopsy strongly affect these numbers.

Adjustment of copy ratios and copy-numbers for purity and ploidy is discussed in Zack et al(120). In this paper, the algebra is incorrect in the q(x)/T factor in the equation for computing R'(x) on page 13. The corrected algebra is:

$$
\begin{aligned}
R'(x) &= q(x)/T = DR(x)/aT - 2(1-a)/aT = (aT + 2(1-a))R(x)/aT - 2(1-a)/aT \\
&= R(x) + 2(1-a)R(x)/aT - 2(1-a)/aT \\
&= [aTR(x) + 2(1-a)R(x) - 2(1-a)]/aT
\end{aligned}
$$

where:

$R'(x)$ = adjusted coverage ratio
$R(x)$ = raw coverage ratio
$q(x)$ = integer copy-number in cancer cells
D = average ploidy across all cells of tumor (of sample)
a = purity
T = tumor ploidy

The corrected equations were incorporated at numerous places into the PureCN CNV analysis presented above. Using R language, the adjustment of a raw copy ratio CRraw for estimated purity and ploidy to produce corrected copy ratio CR was:

```
CR = (purity*ploidy*CRraw + 2*(1-purity)*CRraw - 2*(1-purity))/(purity*ploidy)
ind = (CR < 0)
CR[ind] = 0
```

The inverse operation was also used at times:

```
CRraw = (purity*ploidy*CR + 2*(1-purity))/(purity*ploidy + 2*(1-purity))
ind = (CRraw < 0)
CRraw[ind] = 0
```

Variant allele frequencies (VAFs) are also altered by purity and ploidy. If a given locus has a copy-number (in the tumor cells) of CN and the actual VAF is called VAF, then each tumor cell produces CN*VAF copies of the variant allele. For N cells total, the number of variant allele copies will be CN*VAF*purity*N, since only purity*N of the cells are tumor cells. The total number of copies of the allele (wild type and variant) in N cells is (CN*purity*N + 2*(1-purity)*N). The observed (raw) VAF is therefore:

```
VAFraw = (CN*VAF*purity) / (CN*purity + 2*(1-purity))
```

Solving this for VAF gives the formula for correcting a raw VAF for purity, given the VAF and copy-number CN at its locus:

```
VAF = VAFraw * (2*(1-purity)/(CN*purity) + 1)
```

Classifying into molecular subtypes

The TCGA adenocarcinoma paper(13) developed a molecular subtype classification for stomach cancer. The paper did not provide formal methods for testing DNA sequence data from a biopsy in order to classify it, but it did give general guidelines. The four molecular subtypes are prioritized as follows, and their guidelines are:

1. EBV (Epstein-Barr virus): if the biopsy has strong evidence of infection by this virus, it is classified as EBV.
2. MSI (microsatellite instability): otherwise, if the biopsy was determined to have microsatellite instability, it is classified as MSI.
3. CIN (chromosome instability): otherwise, a biopsy with significant aneuploidy was classified as CIN, and chromosomal arms were considered altered if at least 66% of the arm was lost or gained with a log2 copy-number change greater than 0.1.
4. GS (genomically stable): otherwise, the biopsy was classified as genomically stable.

A custom R program named classifyMolecular.R was developed to apply this classification scheme to the biopsies. The specific algorithms used were:

1. EBV: the count of the number of reads mapping to EBV genes in the panel was adjusted for biopsy purity by dividing the count by the purity estimate from PureCN, for each biopsy. The total read count for each biopsy was likewise adjusted. The purity-adjusted EBV read count for each biopsy was adjusted for library size by multiplying it by the mean of the purity-adjusted total read count and dividing by the purity-adjusted total read count in that biopsy. The resulting normalized and purity-adjusted EBV read counts were clustered into two clusters using k-means. The midpoint between the clusters was chosen as a threshold for calling EBV infection and EBV molecular subtype.
2. MSI: the output file from the MSIsensor program was read and biopsies whose MSIsensor score exceeded the threshold of 3.5 were called MSI molecular subtype.
3. CIN: the output file from GetChrCNV.R was read and for each biopsy the number of altered chromosome arms was counted. The counts were clustered into two clusters using k-means. The midpoint between the clusters was chosen as a threshold for calling CIN molecular subtype for a biopsy. The threshold of 66% of the arm lost or gained was used to call an arm altered, as with TCGA. Unlike TCGA, a loss or gain segment was considered to be any segment whose purity/ploidy-adjusted copy-number was > ploidy+0.75 or < ploidy-0.75 (whereas TCGA used 1.07 (= 2^0.1) instead

of 0.75). This was because a typical single-chromosome gain or loss will change copy-number by exactly 1.0 in the ideal situation, but since the copy-number values are noisy, such a value could easily fall slightly above 1.0 even though the actual value is indeed 1.0. The TCGA value of 1.07 was considered to be too close to 1.0.

In addition to classifying biopsies for molecular subtype, we classified *individuals* for molecular subtype. The rules were simple:

1. Individual is EBV if any biopsy was classified as EBV.
2. Otherwise, individual is MSI if any biopsy was classified MSI.
3. Otherwise, individual is CIN if any biopsy is CIN.
4. Otherwise, individual is GS.

Following this classification process, manual examination of the results revealed some cases of questionable classification. We added arguments to classifyMolecular.R to allow manual override of its automatic classifications. We made the following manual overrides:

- I_20447_S_26976 (T1): sample was forced to be type MSI. Its MSIsensor score was 1.39, below the threshold of 3.5; its sister tumor I_20447_S_26977 (T2) had a score of 10.66. The two tumors had a similar number of somatic variants.
- I_26171_S_26816 (T1): sample was forced to be type GS. It was called as type CIN with an altered chromosome arm count of 11, a little above the threshold of 9.5; its sister tumor I_26171_S_26817 (T2) was 8, and both tumors had a very low number of somatic mutations.

H. pylori infection assessment

Assessment for *H. pylori* infection was done by classifyMolecular.R in a similar manner as for EBV infection. The count of the number of reads mapping to *H. pylori* genes (without regard to the strain(s) of *H. pylori* the reads mapped to) in the panel was adjusted for biopsy purity by dividing the count by the purity estimate from PureCN, for each biopsy. The total read count for each biopsy was likewise adjusted. The purity-adjusted *H. pylori* read count for each biopsy was adjusted for library size by multiplying it by the mean of the purity-adjusted total read count and dividing by the purity-adjusted total read count in that biopsy. The resulting normalized and purity-adjusted *H. pylori* read counts were clustered into two clusters using k-means. The midpoint between the clusters was chosen as a threshold for calling *H. pylori* infection.

Mutation druggability gene list construction

Two lists of genes with drugs approved for targeting specific mutations were used to estimate the potential druggability of the mutations we found. The OncoKB(121) gene list(122) was trimmed by excluding entries with *drug-resistance* levels-of-evidence and removing entries not annotated with a gene targeted by our panel, leaving 50 of the original 64 genes within OncoKB levels-of-evidence 1 through 4. The second gene list is of 69 FDA GC-targeted therapy genes(123), of which 44 were in our panel. The combined list contained 65 genes, and after two on a sex chromosome and five with poor coverage were removed, 58 druggable genes remained on our list.

Mutation signature analysis

Mutation signatures have been identified de-novo in TCGA cancer data from multiple cancer projects, using non-negative matrix factorization(124,125), including signatures specific to GC(126). The original signatures were single-base-pair substitutions with a single base on each side as context. Subsequently, a new study added new single-base signatures and expanded the signatures to double-base and indel substitutions(127). Both original (V2.0) and newer (V3.2) signatures have been curated in the COSMIC Mutational Signatures database(113). Each signature is defined by a probability vector whose sum is 1 and whose elements are the probabilities that the mutational process active for that signature will create a mutation of a given type. For single base substitutions, there are 96 possible mutation types including a single context base on each side, and so the vector has 96 probabilities in it. The total number of signatures in the V2 set is 30 and in the V3.2 set is 78. De novo signature discovery requires a large number of samples in order to pick up the signals of different signatures accurately. When the number of available samples is small, as it is in this MSEQ project, de novo signature discovery cannot be done.

Signatures operating in single samples can be estimated by multiple linear regression techniques to find the combination of weights of all signatures under consideration that minimizes the error in predicted mutation spectrum vs. actual mutation spectrum. We used one such algorithm is deconstructSigs(128), an R package, to estimate signature weights. The algorithm requires a sufficient number of mutations in order to perform well, and because our project used a panel with limited genome coverage, the mutation count per sample was too low to expect high-quality results. Instead of extracting per-sample signature weights, we combined mutations separately for MSS samples and MSI samples, then ran deconstructSigs on the combined mutation set, to estimate signature weights of signatures caused by mutational processes presumably operating within the set of samples. We used the V3.2 signature set, but removed several signatures from it:

a) Sequencing artifact signatures: SBS27, SBS43, SBS45-60

b) Signatures with unknown etiology: SBS8, SBS12, SBS16, SBS17a, SBS19, SBS23, SBS28, SBS33, SBS34, SBS37, SBS39-41, SBS89, SBS91, SBS93, SBS94

c) Signatures not expected to occur within our GC tumors: SBS7a-d (UV exposure), SBS11 (alkylating agent temozolomide), SBS25 (chemotherapy treatment), SBS29 (tobacco chewing), SBS31 (platinum chemotherapy), SBS32 (azathioprine treatment), SBS35 (platinum chemotherapy), SBS38 (UV exposure), SBS87 (thiopurine chemotherapy treatment)

d) Certain signature numbers do not exist as signatures: SBS61-83

The 31 signatures used in the final signature analysis were: SBS1-6, SBS9, SBS10a-d, SBS13-15, SBS17b, SBS18, SBS20-22, SBS24, SBS26, SBS30, SBS36, SBS42, SBS44, SBS84-86, SBS88, SBS90, SBS92.

Phylogenetic analysis

Existing programs for printing and annotating trees were found to be very inadequate for our needs, so a custom R program named plotTrees.R was developed to generate, annotate, and print phylogenetic trees. **Supplementary figure 2** shows a selected subsample of trees plotted by this program. Each tree corresponds to one individual and has one leaf node for each biopsy of the individual (including the normal biopsy). Also, there are four supplementary figures containing phylogenetic trees for every patient (**Supplementary figures 3, 5, 6 and 8).**

PlotTrees.R uses the R phylogenetic tree packages “ape”(129) and “geiger”(130) to create the trees, and optionally performs bootstrapping on them. It was designed to produce trees from somatic SNV, indel data, and copy-number data of various kinds. It has many options that determine how trees are generated for each biopsy, including whether to use SNV/indel data, CNV data, or both. Three trees can be plotted on one page, a tree made from SNV/indel data, one made from CNV data, and one made from both kinds of data. Different sets of options can be applied to control not only the specific CNV data used to make the trees, but also to control the annotation on the trees. The biggest part of the program involves positioning tree branch annotation to make the tree as uncluttered and readable as possible, and that process is not described here. The plotTrees.R program is not general enough for use outside our lab.

The program computed and plotted one tree at a time, each tree belonging to a single individual. The remaining description applies to creation of one tree, for one individual.

To make a phylogenetic tree, a distance matrix must be created. It is a square matrix with one row and one column for each biopsy, and the values in the matrix represent the similarity of the biopsies of that row and column, with a value of 0 indicating complete dissimilarity and larger values indicating greater similarity. A distance matrix is created by first creating an event matrix, which has one column per biopsy, one row for each event that will be used to determine relative ancestry of the biopsies, and values that may be simply 0 if the biopsy did not have the event and 1 if it did, or the values may be more complex. This is discussed below when the methods for creating each type of tree are described.

After the event matrix is created, it is used to create a distance matrix by applying a distance metric to it. In plotTrees.R the distance metric is the standard Euclidean distance, so that the distance between two biopsies is the sum of the square of the differences between the event matrix columns for those two biopsies. The distance matrix is turned into a standard R distance matrix object with function as.dist(). The phylogenetic tree is then computed with the fastme.bal() function in the “ape” package, which performs the “minimum evolution” algorithm(131) and the root() function is used to root the tree at the normal biopsy.

When plotTrees.R is configured to perform bootstrapping, the boot.phylo() function is called with argument x being the transpose of the event matrix and the argument FUN being a function that recomputes a new tree from a truncated event matrix using the same method as was used for the main tree. The bootstrap result can be annotated on the tree branches as a percent number, which is derived as 100*prop.clades()/numBootstraps, where prop.clades() is the “ape” package function that returns the number that was associated with each tree node by the boot.phylo() function. When bootstrapping is performed, numBootstraps was always 100, i.e. 100 rounds of bootstrapping were performed.

Somatic SNV/indel-based trees

The somReliable filter was used to filter somatic variants to obtain reliably-called variants (both SNVs and indels) from which to compute trees. (The more stringent somCodSplNoSyn filter was used to select variants for annotating onto the trees.) The event matrix was created by examining the VarStat variable for the variants passing the somReliable filter and for the tumor biopsies of the individual. Each biopsy with a WT value in VarStat, and the normal biopsy, were assigned an event matrix value of 0. Those with a SOM value were assigned a value of 1. Those with an LOH value were assigned a value of 2, so that LOH events are weighted twice as much as non-LOH events. SOM and LOH are treated as the *same* event, even though

the LOH may have occurred as two separate events (a SOM event and then another mutation causing LOH). Treating them as separate events was problematic and may not be the actual situation anyway.

CNV-based trees

Several different types of CNV data could be used as events for creating an event matrix:
1. Change in ploidy from the diploid ploidy state
2. Chromosome and chromosome arm copy-number changes
3. Segment edge copy-number changes
4. Gene copy-number changes and loss-of-heterozygosity

plotTrees.R allows any subset or all of these data to be used to create the event matrix.

Ploidy events are determined by data in the PureCN QC file, which contains the purity and ploidy estimates of PureCN (curated). Events are entered into the event matrix as one or two rows. Ploidy gain events are treated separately from ploidy loss events. The actual ploidy value was not used beyond comparing it to 2 to determine loss or gain, because the precise value may be unreliable depending on how well PureCN is able to determine the correct purity/ploidy solution. If any tumor biopsy had a ploidy gain, a row was added to the event matrix with all biopsies having 0 except those having the ploidy gain being 1. Likewise, a row was added if any tumor biopsy had a ploidy loss.

Chromosome and chromosome arm copy-number change events are determined by data in the chromosome CNV file containing the output of the GetChrCNV.R program described earlier, specifically by the values isGain, isLoss, isAltered, and isLOH for each chromosome arm and the chromosome as a whole. When one or more of these flags is set for a chromosome in any tumor biopsy of the individual, events are created in the event matrix. Each chromosome can have one or more of these events:
1. whole chromosome gain
2. whole chromosome loss
3. whole chromosome LOH
4. chromosome p arm gain
5. chromosome p arm loss
6. chromosome p arm altered
7. chromosome p arm LOH
8. chromosome q arm gain
9. chromosome q arm loss
10. chromosome q arm altered
11. chromosome q arm LOH

Each of these is treated as a separate event. For example, if one biopsy has "whole chromosome isGain" and another has "whole chromosome isLoss", these are separate events, even though the chromosome is the same. A single event can occur in any subset of biopsies or all of the biopsies. For example, biopsies 1 and 2 might have a "whole chromosome #21 gain" while 3 and 4 might have "whole chromosome #21 loss".

The chromosome arm gain events are suppressed if the whole chromosome gain event is present, and likewise for losses. The whole chromosome altered event doesn't exist because it is likely in that case that one arm will have a gain and the other arm will have a loss, or one or both arms will be altered, so the event is picked up in the arms.

The meaning of "isAltered" is that a significant part of the chromosome or arm has either a gain or a loss (gain and loss distance is summed). That is consistent with TCGA definition of chromosome alteration used for assigning chromosome instability to a sample.

The event matrix contains 0 when a biopsy did not have the event, and 1 when it did.

Segment edge copy-number change events are determined by data in the CNV edges file containing the output of the TestCNVedgesOfSamplesOfPerson.R program described earlier. Segment edge data is used for these events, rather than creating events for each *segment* that is identified as having a copy-number change, because segments do not necessarily correspond to single ancestral CNV events. If a second copy-number change occurs in one biopsy in the middle of a segment in which an ancestral copy-number change occurred that was inherited by all the biopsies, the biopsy with two events will have three segments rather than one. It is unlikely that the actual ancestral order of events could be determined from the segment data. Therefore, we took the approach of recognizing that each ancestral segment edge should be present in all biopsies even if each segment were not. By testing the copy-number to the left and right of each segment edge (regardless of which biopsy it occurred in), the edge can be validated as being present, with a p-value attached to the test. The

TestCNVedgesOfSamplesOfPerson.R program performs this edge testing. While it is possible that two CNV events with opposite effects on copy-number will occur at the same location within a biopsy, this is regarded as unlikely and not a significant influence on the phylogenetic results. Also, since every CNV event generates two edges, each event could be entered into the event matrix twice (if both edges are tested as significant). The effect of this could be countered with event matrix weights, discussed below.

Edge events are created from tested segment edges with filter=PASS, SVTCON != INCONSISTENT, and are not near the edge of a chromosome arm (which is defined as being located farther than 500,000 bp away from a chromosome end or centromere). The event occurs only in biopsies with SIG=YES, and the edge is ignored if the DIR value differs among the SIG=YES biopsies. The edge is also ignored if the relationship of CR_L to CR_R is inconsistent with DIR in any SIG=YES biopsy. Two types of events are created, copy-number decrease events and copy-number increase events, and edges are ignored if both types of events occur at the same position, because that is unlikely to have occurred independently.

The event matrix contains 0 when a biopsy did not have the event, and 1 when it did.

Gene-CNV-segment copy-number change and LOH events are determined by data in the gene CNV file containing the output of the CreateCNVgenePvals.R program described earlier. A single copy-number change or loss-of-heterozygosity can alter the copy-number of many genes, even hundreds of genes, depending on its length. Therefore, defining an event for *each gene* that undergoes such a change would completely distort the similarity between different biopsies. To try to avoid this problem, gene-CNV-segment events were implemented in a manner that assigns a single event to multiple genes.

A gene-CNV-segment copy-number gain/loss event is defined as a gene whose “CNVtype”, generated by CreateCNVgenePvals.R as a result of t-tests of gene marker copy ratios, is AMP or DEL, and the purity/ploidy-adjusted copy-number changes from the ploidy value in a direction consistent with “type”, and the PureCN “C.flagged” flag for the gene is not set (which would indicate unreliable copy-number) in any biopsy of the person. Genes not in the target panel are excluded. Since copy-number can go up in one biopsy due to a segment amplification, and down in a different biopsy due to a segment deletion, amplifications and deletions are treated as two separate events, so that one or more biopsies could have an amplification event in a gene, and one or more OTHER biopsies could have a deletion event in the same gene.

A gene loss-of-heterozygosity event is defined as a gene whose “loh” flag has been set by PureCN, and again the PureCN “C.flagged” flag for the gene is not set in any biopsy of the person. Genes not in the target panel are excluded. Loss-of-heterozygosity can occur with or without copy-number gain or loss, so loss-of-heterozygosity is treated as a separate event from copy-number gain/loss, even though in some cases both might be caused by the same underlying CNV event.

The same segment CNV can generate multiple gene copy-number gain/loss events and/or multiple gene loss-of-heterozygosity events (when the segment extends over more than one gene). In order to not count such a segment event as multiple events, for each gene on the segment, adjacent gene events are combined into a single event whenever they occur on the same segments within each biopsy. For example, if genes A and B have an amplification and both of them are located in CNV segment S1 in biopsy 1, segment S2 in biopsy 2, segment S3 in biopsy 3, etc. then they are combined into the same copy-number gain event assigned to both of those genes. If the segment number changes in any biopsy, the gene events are not combined. The same is done for deletions and for loss-of-heterozygosity. This is expected to eliminate the large majority of multiple gene events from the same segment. The actual copy-numbers of the genes and segments do not take part in this process.

It is possible that a gene event is also called as a chromosome arm event, but we do not attempt to distinguish that case, and treat each type of event independently, so in those cases the event will be counted twice.

The actual value of the copy-number of a gene CNV event is ignored, so if two biopsies have an increase in copy-number in a gene, this is considered the same event even if the copy-number is different in the two biopsies. The reason for this is, first, PureCN copy-numbers are not accurate enough that we can be certain that two different copy-numbers reflect actual differences, and second, most likely if the copy-numbers really are different, there were at least two copy-number events, one of them shared by the two biopsies, but we have no way to be sure of that and splitting the event into two events might or might not reflect the actual events. So, to keep it simple, just one event is created. However, the actual delta copy-numbers of the biopsies can be annotated on the tree if desired.

The event matrix contains 0 when a biopsy did not have the event, and 1 when it did.

Event matrix weighting

The event matrix as described above contains only 0's (no event) and 1's (event) except for SNVs/indels with both WT and LOH genotypes, which have 2 for a value. However, there is no reason to think that one type of event should have the same weight in determining similarity, as another type of event. This is a problem that was addressed by creating a weight vector that assigns a weight to each type of event (SNV/indel event or any of the four types of CNV events). The event matrix entries are multiplied by the appropriate weights for each row of the matrix before it is used to generate the distance matrix. The weight of SNVs/indels is always set to 1.0 as a base reference. The relative weights of the CNV events were chosen by simple seat-of-the-pants estimation. The balance of weights between CNV and SNV/indel events was chosen by generating trees using different weight balances and comparing the SNV/indel-only, CNV-only, and SNV/indel/CNV (combined) trees. The final balance was chosen as the one whose combined trees appeared to have a topology midway between the SNV/indel and CNV trees:

1. SNVevents=1.0
2. Ploidy=0.5
3. ChrCNVs=1.0
4. CNVedges=0.75
5. CNVgenes=0.5

No algorithm has been found that might let us choose an event weight vector in an unbiased manner, and therefore the combined trees are perhaps to be trusted less than the other two tree types.

When plotTrees.R options are set so as to annotate a particular type of mutation event on the tree, the list of such events is analyzed, one event at a time, to determine on which branch or branches the annotation should be placed, starting at the root node corresponding to the normal biopsy, and moving down the tree towards the leaves. On a particular branch, if all biopsy leaf nodes downstream of that branch have the mutation in question, then that event is annotated on that branch. Otherwise, the same analysis continues on one or both downstream branches, depending on whether any leaf nodes of one or both branches have the mutation. Therefore, it is possible for the same event to be annotated on multiple branches. For example, if a tree has three biopsies, T1, T2, and T3, and T1 branches off first, then if T1 and T2 but not T3 had a certain mutation event, then the event would be annotated on the branches leading to T1 and T2 but not on the branch leading to T3. If on the other hand the event were clonal and occurred in all three biopsies, it would be annotated on the branch leading out of the normal biopsy, because that is the branch that is upstream from all three biopsies. When a branch is annotated with an event, the inference is that the event occurred somewhere along that branch in the ancestry of the leaf node biopsies of that branch. The order of occurrence of events annotated on a branch is not inferred and remains unknown.

Supplementary figures

The following pages show supplementary figures referenced from the manuscript and within this supplementary document.

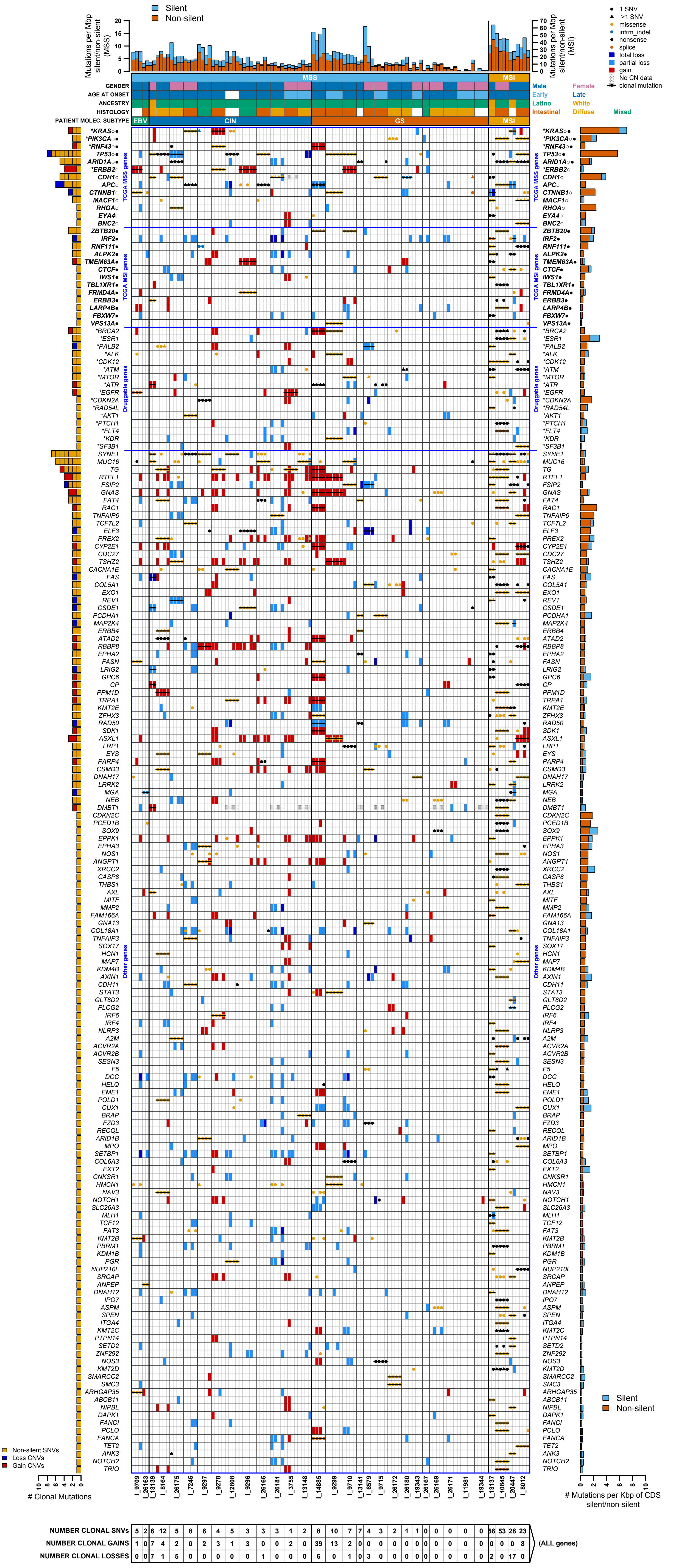

Supplementary figure 1: Genes with clonal SNV mutations in at least one gastric cancer MSS or MSI patient

Genes shown on both left and right sides are those where at least one patient has a clonal somatic non-silent single nucleotide or short indel (SNV) mutation. Genes are divided into three sections: TCGA MSS genes are those genes found to be gastric cancer drivers in non-hypermutated samples by the TCGA GC study; TCGA MSI genes are similar but for TCGA hyper-mutated samples and not already shown in the TCGA MSS section; Druggable genes are genes previously identified as druggable and not already shown in the TCGA sections; and Other genes are all remaining genes not falling into one of those groups. Patients (I_#### IDs at bottom) are separated by a dark vertical line and patient biopsies within them are separated by a lighter line. SNV and CNV mutations are shown for each gene/biopsy combination. Gains and losses are distinguished by box color, while SNVs are shown as a filled circle or triangle using color to distinguish the mutation type (key at upper-right; nonsense mutations include stop gain/loss and frameshift). A triangle rather than a circle means a gene has more than one mutation in a patient, and the type shown is the one generally most detrimental. A bar through all biopsies means the mutation is clonal. The left side bar plot shows the total number of clonal mutations of each type in each gene, with short vertical lines separating individual patients. Numbers in boxes at the bottom give the total number of clonal mutations of each type in each patient in all genes (not just the ones shown). Patient molecular subtypes (EBV, CIN, GS, MSI) are indicated by color-coded bars just above the main figure area, and above those are additional color-coded bars indicating patient MSS/MSI status, sex, age of onset of gastric cancer (where late onset was defined as occurring at age > 50), ancestry (Latino or white), and tumor histology. The top bar plot shows SNV mutation rate in each biopsy, in the region targeted by the panel, with non-silent and silent rates distinguished by color. MSS patients have a lower mutation rate and use the y-axis scale on the left, while MSI patients with a higher rate use the right-side scale. The right side bar plot shows the SNV mutation count in each gene summed over all 115 biopsies, normalized by dividing by the gene CDS length in Kbp, with non-silent and silent mutations distinguished by color. Gene names are in bold when they were found to be gastric cancer drivers by the TCGA GC study, with an open circle denoting driver genes in non-hypermutated TCGA samples and a closed circle denoting those in hypermutated samples.

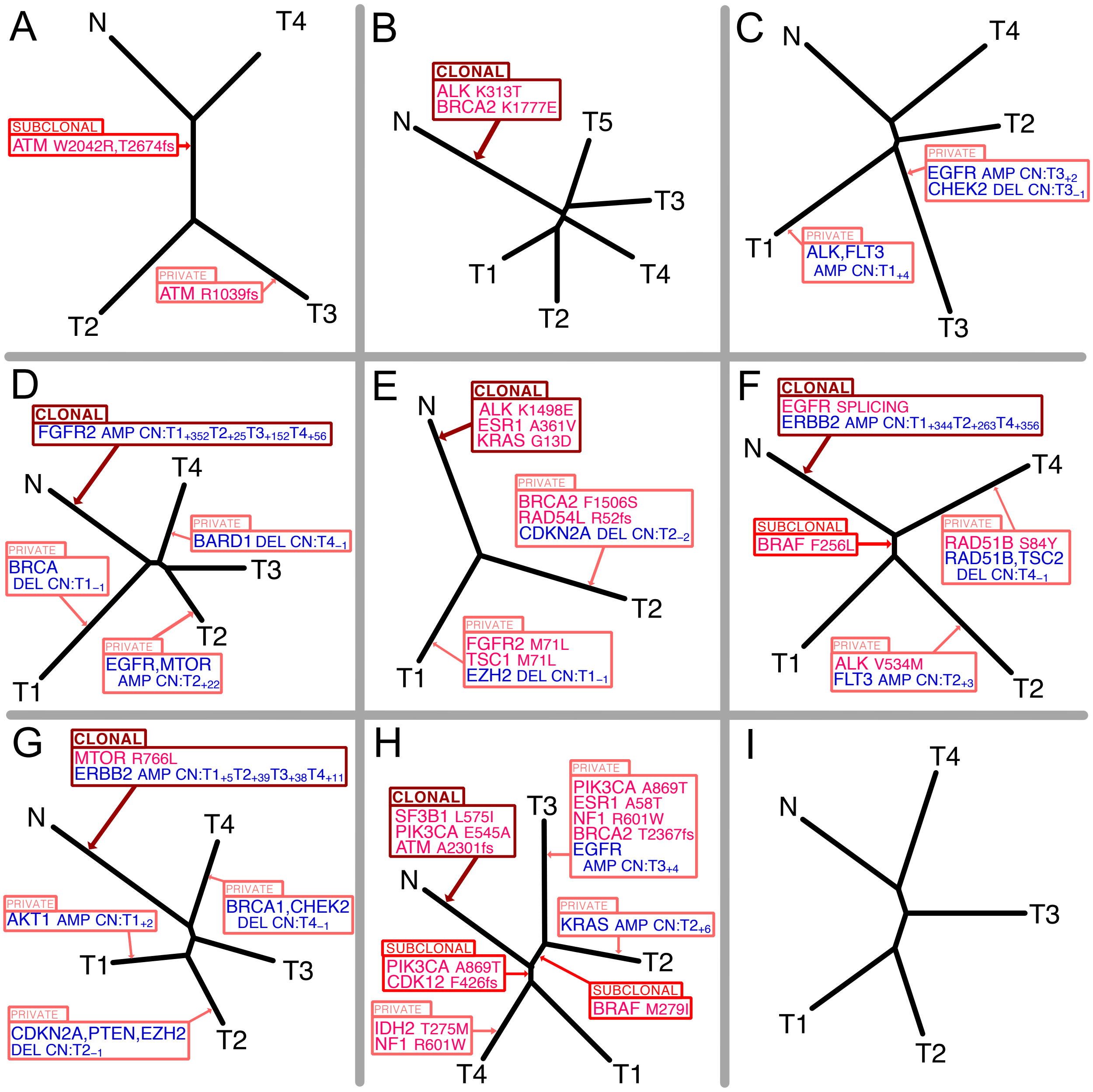

Supplementary figure 2: Phylogenetic trees depicting inferred ancestral relationships of biopsies.

Each tree shows one patient's biopsies, and each tree is made from a distance matrix derived from an event matrix of all SNV and indel mutations identified using our cancer gene panel (CNV changes are not used for the event matrix). Leaves are marked with the biopsy ID, with "N" for the normal biopsy and "T#" for tumor biopsies, and trees are rooted at the normal biopsy. Tree edge length is proportional to the total weight of mutations occurring on the edge, and the preponderance of private over clonal and subclonal mutations leads to most terminal edges being longer than internal ones. Tree edge annotations show genes containing a non-silent SNV, indel, or CNV mutation occurring on the edge and that were identified as having an approved drug (OncoKB) that targets gene mutations (not necessarily the ones occurring here; these mutations include all non-silent mutations, not just those identified as pathogenic in the main manuscript). Internal edges correspond to subclonal mutations, present in multiple biopsies. **Mutations on the edge leading from N are clonal mutations.** Red text describes SNV and indel variants with amino acid changes (fs: frameshift) and blue text describes CNV gains and losses (AMP: amplification or copy-number gain, DEL: deletion or copy-number loss; CN: estimated gene copy-number changes as subscripts of tumor IDs). **A.** SNV mutations in druggable genes, such as *ATM*, are poorer drug targets if they are private (R1039fs in T3) or subclonal (W2042R and T2674fs in T2 and T3). **B.** Clonal druggable mutations (those occurring in all biopsies), such as *ALK* (K313T) and *BRCA2* (K1777E), make better targets. The lack of subclonal and private mutations accompanying clonal mutations seen here is rare. **C.** The same situation is true for copy-number mutations, such as the *EGFR, ALK* and *FLT2* amplifications and CHEK2 deletion, which are private mutations. **D.** The *FGFR2* amplification is clonal while other mutations are private. Clonal druggable mutations are seen in only about half of all CIN molecular subtypes. **E, F, G**. Mixtures of druggable SNV and CNV mutations, of all three classes (private, subclonal, and clonal), is common, making it likely that a gene chosen from a single biopsy will be an ultimately ineffective drug target. It is also common to have more than one druggable clonal mutation, which can provide options for selecting the better drug or the use of a drug combination. **H.** Sometimes a larger number of clonal and non-clonal druggable mutations are present, most often seen with the MSI molecular subtype. **I.** In some patients no druggable mutations are found, clonal or non-clonal, very common within the GS molecular subtype.

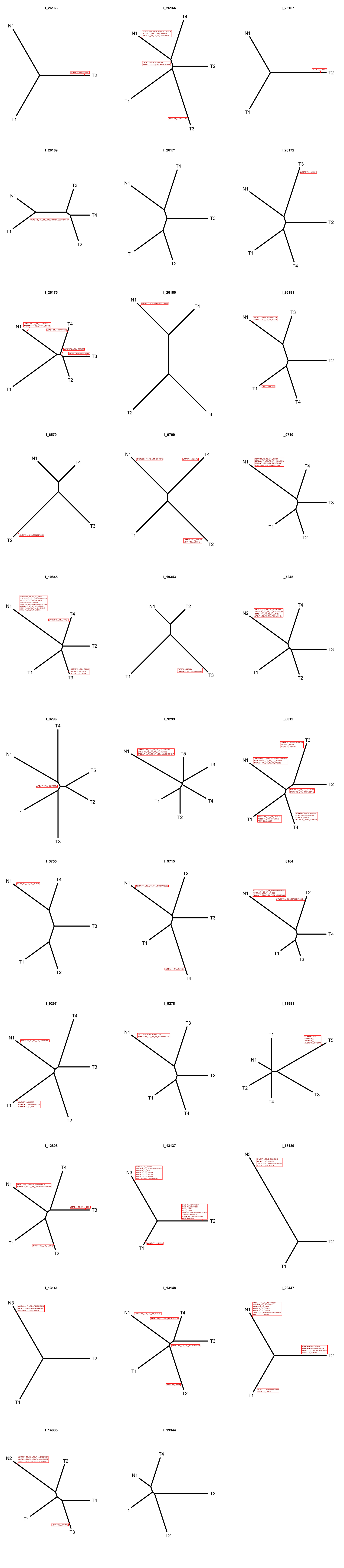

Supplementary figure 3: Phylogenetic trees for mutations in Figure 1
Phylogenetic trees, one per patient, generated to visualize the evolution of each patient's tumor. The event matrix used to create the tree is generated from SNV and indel mutations. Tree branches are annotated with mutations occurring on that branch within one of the genes shown in **Figure 1**. The node marked N1 or N2 represents the germline state of the patient's genome, while T1, T2, etc. are the tumor biopsies. Red text describes SNV and indel variants including mean variant allele frequencies (adjusted for purity/ploidy) across biopsies and amino acid changes, and blue text describes copy number changes including number of copies gained or lost. Genes found by the TCGA gastric cancer study to be drivers are shown in bold, with ○ denoting driver genes in non-hypermutated TCGA samples and ● denoting those in hypermutated samples.

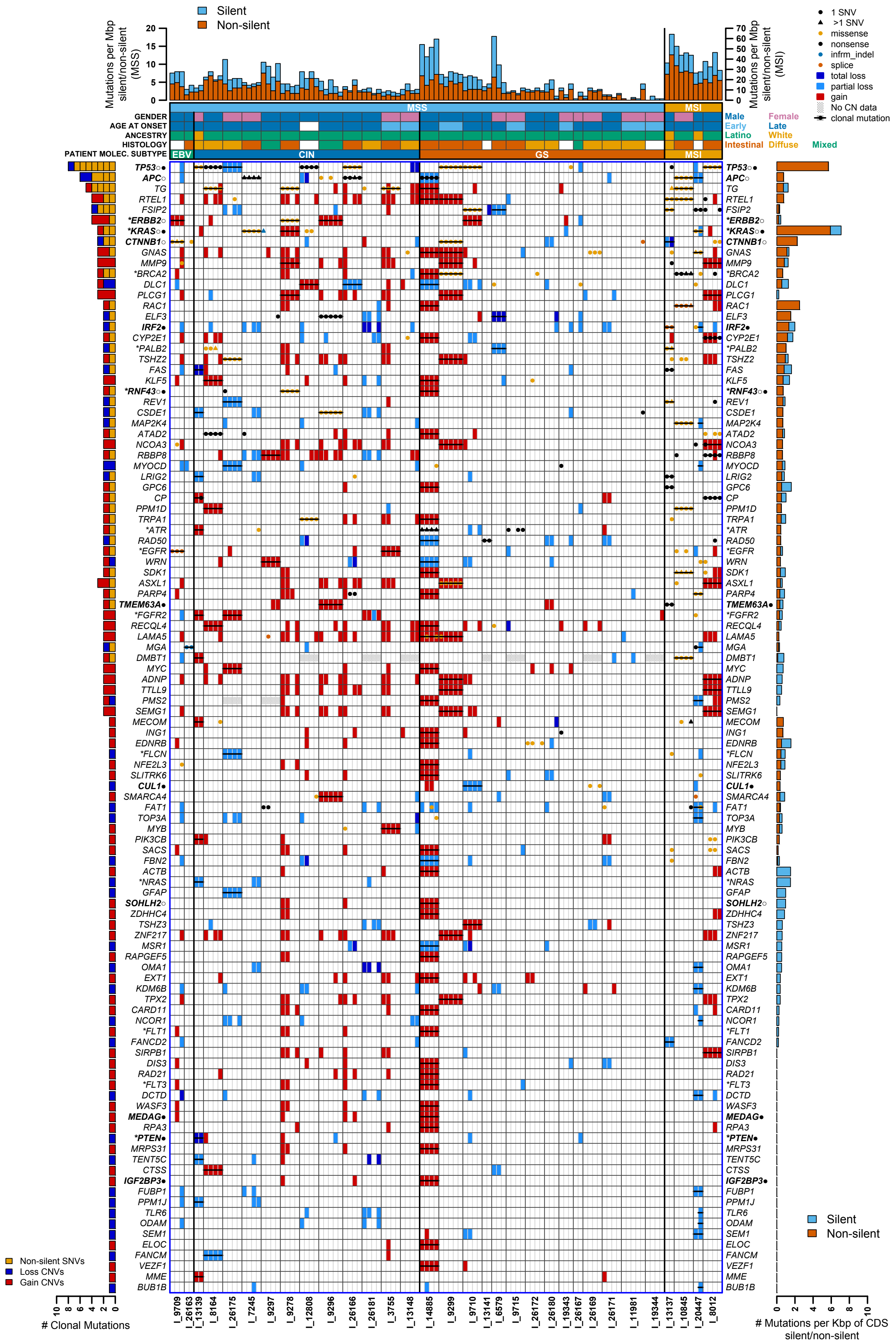

NUMBER CLONAL SNVs	5	2	6	12	5	8	6	4	5	3	3	3	1	2	8	10	7	7	4	3	2	1	1	0	0	0	0	0	56	53	28	23	(ALL genes)
NUMBER CLONAL GAINS	1	0	7	4	2	0	2	3	1	3	0	0	2	0	39	13	2	0	0	0	0	0	0	0	0	0	0	0	0	0	0	8	
NUMBER CLONAL LOSSES	0	0	7	1	5	0	0	0	0	0	1	0	0	0	6	0	1	0	3	0	0	0	0	0	0	0	0	0	2	0	17	0	

Supplementary figure 4: Genes with clonal CNV mutations in at least one gastric cancer MSS or MSI patient

Gene names shown are those in our druggable gene list where at least one patient has a clonal somatic non-silent single nucleotide or short indel (SNV) or copy number gain/loss (CNV) mutation. Panel layout is identical to Supplementary figure 1.

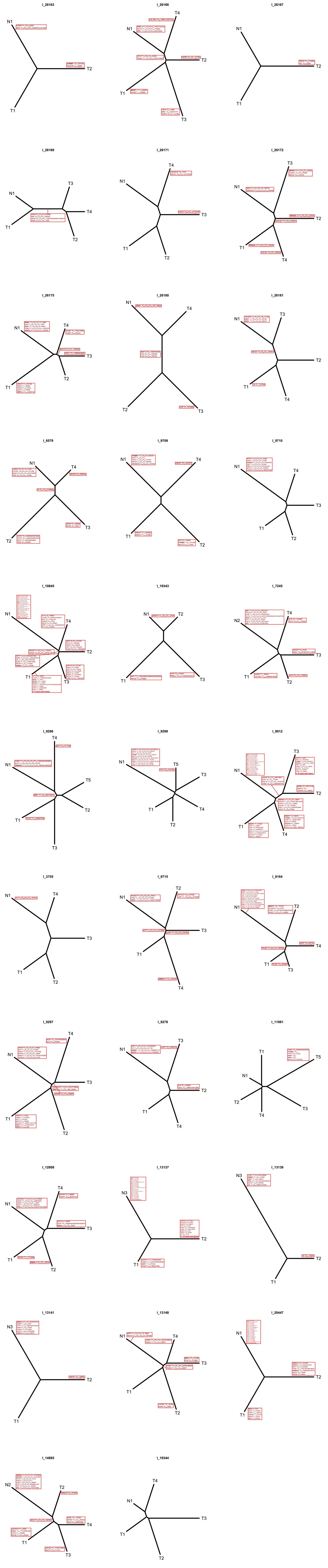

Supplementary figure 5: Phylogenetic trees for mutations in Supplementary figure 1
Like Supplementary figure 3, except the tree branches are annotated with mutations in one of the genes shown in **Supplementary figure 1**.

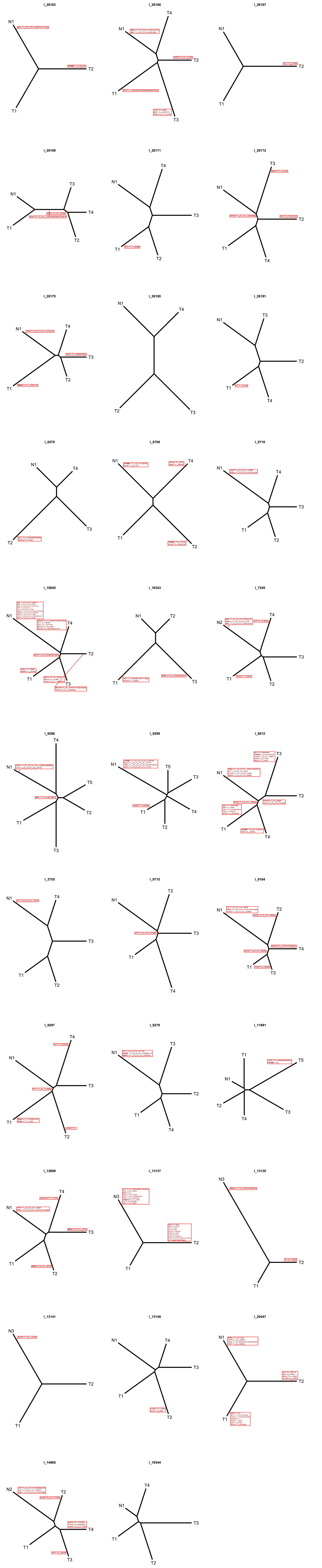

Supplementary figure 6: Phylogenetic trees for mutations in Supplementary figure 4
Like Supplementary figure 3, except the tree branches are annotated with mutations in one of the genes shown in **Supplementary figure 4**.

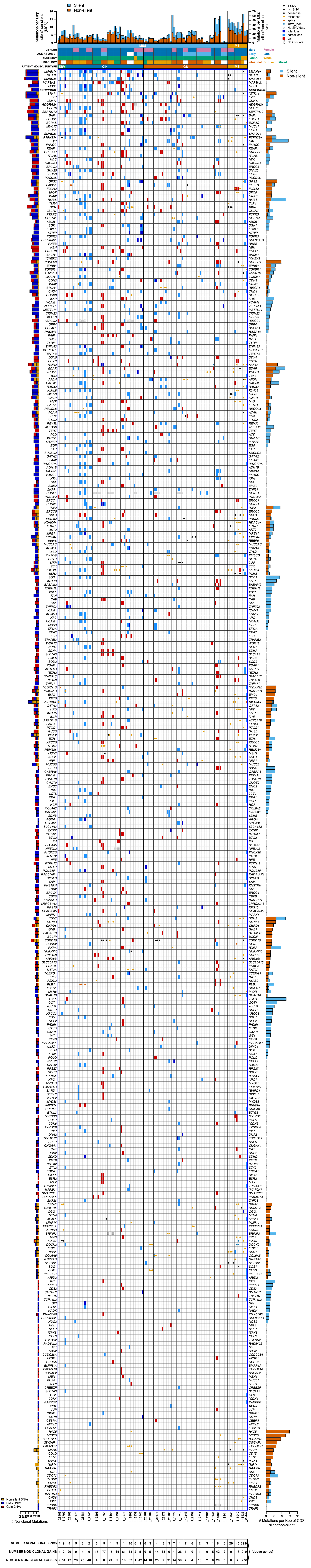

	I_26163	I_9709	I_26166	I_26175	I_26181	I_7245	I_9296	I_3755	I_8164	I_9297	I_9278	I_12808	I_13139	I_13148	I_26167	I_26169	I_26171	I_26172	I_26180	I_6579	I_9710	I_19343	I_9299	I_9715	I_11981	I_13141	I_14885	I_19344	I_10845	I_8012	I_13137	I_20447
NUMBER NON-CLONAL SNVs	4	9	4	5	3	2	5	5	4	9	1	10	0	1	0	3	4	3	3	7	1	7	6	2	3	1	8	0	29	45	39	9
NUMBER NON-CLONAL GAINS	4	2	20	0	4	0	17	77	15	14	61	14	2	5	0	5	14	13	6	1	26	1	0	1	0	5	42	2	0	18	0	0
NUMBER NON-CLONAL LOSSES	9	51	17	29	75	46	4	8	24	5	18	67	1	42	14	10	25	7	31	14	58	7	4	13	2	0	20	5	0	7	2	50

(above genes)

Supplementary figure 7: Genes with only non-clonal mutations in all gastric cancer MSS and MSI patients

Gene names shown are those where no patient has a clonal somatic non-silent single nucleotide or short indel (SNV) or copy number gain/loss (CNV) mutation, but at least one patient has a non-clonal mutation of one of those types. Panel layout is identical to Supplementary figure 1 with the exception that the boxed numbers at the bottom are clonal mutation counts for the shown genes only.

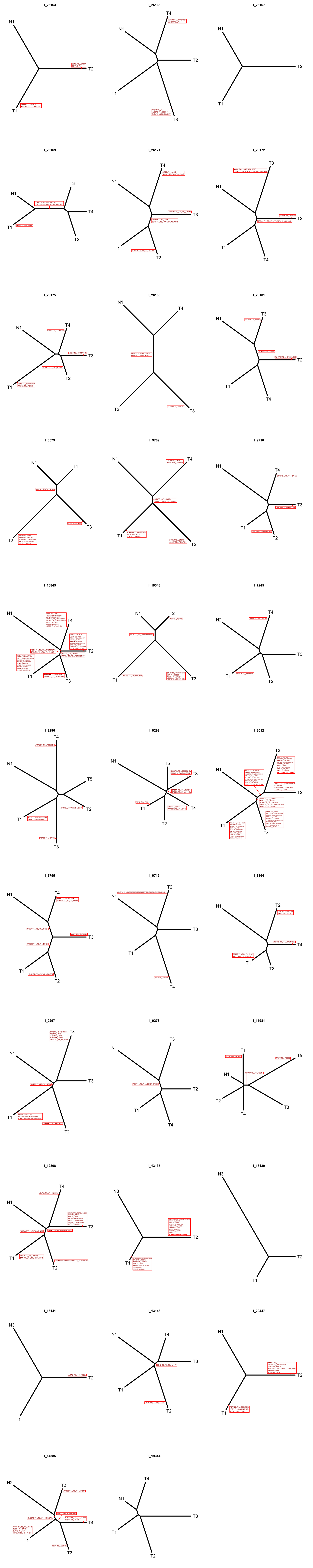

Supplementary figure 8: Phylogenetic trees for mutations in Supplementary figure 7
Like Supplementary figure 3, except the tree branches are annotated with mutations in one of the genes shown in **Supplementary figure 7**.

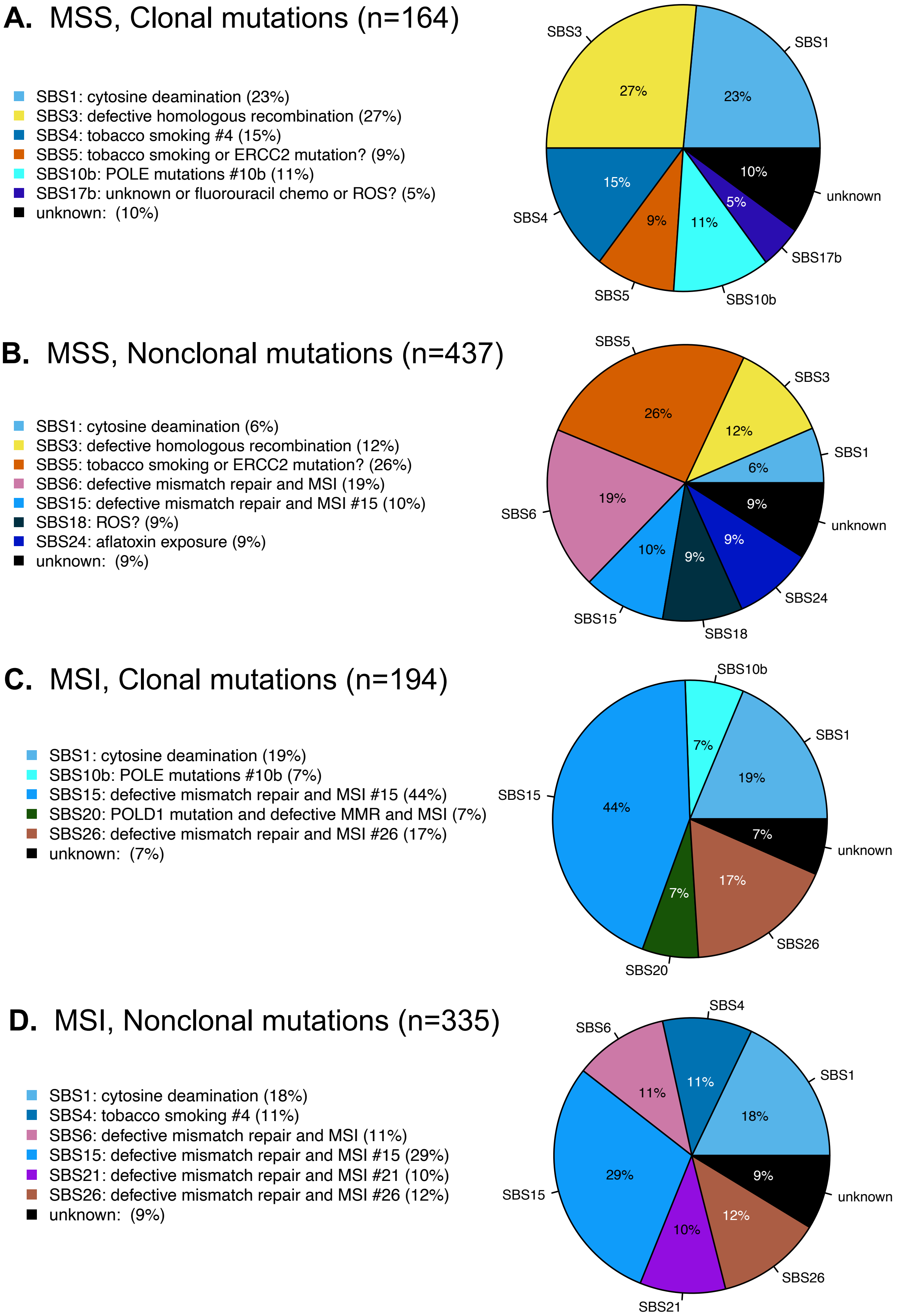

Supplementary figure 9: Mutation signatures found in clonal and nonclonal mutations in MSS and MSI samples

A. Mutation signature weights found for the combined set of clonal mutations in MSS samples. **B.** For nonclonal mutations in MSS samples. **C.** For clonal mutations in MSI samples. **D.** For nonclonal mutations in MSI samples.

Supplementary tables

Supplementary table 1. DNA repair genes that were somatic pan-cancer panel gene candidates.

Preferred Symbol	Alternate Symbol	Preferred Symbol	Alternate Symbol
ADH1B		MUS81	
ALDH2		NBN	
ATM		PALB2	
ATR		PARPBP	
ATRIP		POLE	
BACH1		POLH	
BCCIP		RAD50	
BLM		RAD51AP1	
BRAP		RAD51B	
BRCA1		RAD51C	
BRCA2		RAD51D	
BRIP1		RAD52	
EMSY	C11orf30	RAD54B	
CAT		RAD54L	
CYP2E1		RAD54L2	
DNA2		UIMC1	RAP80
EME1		RBBP8	
EME2		RECQL	
ERCC1		RECQL4	
ERCC4		RECQL5	
EXO1		REV1	
FAN1		REV3L	
FANCA		RMI1	
FANCB		RMI2	
FANCC		RPA1	
FANCD2		RPA2	
FANCE		RPA3	
FANCF		RPA4	
FANCG		RTEL1	
FANCI		SHFM1	
FANCL		SOD1	
FANCM		SRCAP	
FEN1		SWSAP1	
HELQ		SYCP3	
INIP		TOP3A	
KEAP1		WRN	
MAD2L2		XRCC2	
MRE11A		XRCC3	
MORF4L1	MRG15	ZRANB3	
POLD1			

Supplementary table 2. Genes whose CDS is less than 90% covered by the somatic pan-cancer panel.
Abbreviations: CDS=Coding Sequence

Gene	CDS Coverage	Comments
ARID1B	89%	Exon 1 has 3 regions totaling about 350 bp that aren't covered
CD1D	89%	Exon 3 longer in 'known genes' than 'RefSeq genes', longer not covered
RAD51C	89%	One exon in 'known genes' but not 'RefSeq genes', not covered
HLA−DRB1	88%	
OTUD7A	88%	
RPL5	88%	
FOXQ1	88%	
RAD51B	86%	Some small exons present in some isoforms not covered
SMARCB1	83%	
PHOX2B	81%	
CEBPA	81%	
RUNX1	80%	
STK19	80%	
CD70	80%	
MORC4	77%	
PRSS1	76%	
TNF	75%	
FRG1BP	75%	
PDGFRA	74%	
JUP	74%	
HLA−B	69%	
RXRA	62%	
GPI	59%	
TNFRSF14	53%	Several exons present in 'known genes' and one in 'RefSeq' not covered

Supplementary table 3. Molecular subtype patient count comparison with TCGA.
Abbreviations: MSEQ=this multiregional sequencing project, TCGA=The Cancer Genome Atlas, EBV=Epstein-Barr Virus, MSI=Microsatellite Instability, CIN=Chromosomal Instability, GS=Genomically Stable

	MSEQ		TCGA		
Type	All Patients	Latino Patients	All Patients	White Patients	Asian Patients
EBV	2 (6.2%)	2 (7%)	26 (8.8%)	19 (8%)	8 (11%)
MSI	4 (12.5%)	2 (7%)	64 (21.7%)	42 (18%)	15 (20%)
CIN	12 (37.5%)	11 (38%)	147 (50%)	146 (61%)	38 (51%)
GS	14 (43.8%)	14 (48%)	58 (19.7%)	31 (13%)	13 (18%)
Total	32	19	295	238	74

Supplementary table 4: Mutation signatures by molecular subtype, histology, and clonality in MSEQ vs. TCGA.
MSI=microsatellite instability, MSS=microsatellite stable, CIN=chromosomal instability, GS=genomically stable, MSEQ=this project, TCGA=The Cancer Genome Atlas, SBS=single base substitution, HR=homologous recombination, MMR=mismatch repair, POLE=DNA Polymerase Epsilon, ROS=reactive oxygen species, POLD1= DNA Polymerase Delta 1.

Signature	Proposed etiology	MSI (%)		MSS (%)		CIN (%)		GS (%)		MSEQ		TCGA		MSEQ Latino MSS Patients (%)		TCGA White/Asian MSS Patients (%)
		Clonal	Non-clonal	Clonal	Non-clonal	Clonal	Non-clonal	Clonal	Non-clonal	GS Diffuse	GS Intestinal	GS Diffuse	GS Intestinal	Clonal	Non-clonal	All Mutations
# Patients		4	4	28	28	12	12	14	14	5	5	39	13	28	28	225
Total # Mutations		194	335	164	437	77	197	72	208	60	181	243	81	164	437	1984
SBS1	Spontaneous deamination	19	17	24	7	38	18	14	-	13	-	31	29	24	7	19
SBS3	HR deficiency	-	-	29	18	21	-	26	-	-	18	-	-	29	18	-
SBS4	Tobacco carcinogens	-	12	15	-	8	-	7	6	-	-	10	6	15	-	9
SBS5	Aging related	-	-	8	23	14	35	11	30	25	20	32	32	8	23	32
SBS6	MMR deficiency	-	12	-	19	-	-	-	-	-	9	-	16	-	19	-
SBS10b	POLE mutations	7	-	12	-	-	-	21	-	29	-	11	-	12	-	14
SBS15	MMR deficiency	44	30	-	10	-	16	-	26	6	15	-	-	-	10	9
SBS17b	Unknown etiology	-	-	-	-	8	-	-	-	-	-	7	-	-	-	11
SBS18	ROS damage	-	-	-	9	-	-	-	7	-	12	-	7	-	9	-
SBS20	POLD1 mutations	7	-	-	-	-	-	-	-	-	-	-	-	-	-	-
SBS21	MMR deficiency	-	10	-	-	-	-	-	-	-	-	-	-	-	-	-
SBS24	Aflatoxin exposure	-	-	-	11	-	15	14	16	17	17	-	-	-	11	-
SBS26	MMR deficiency	18	12	-	-	-	-	-	-	-	-	-	-	-	-	-
-	Other/ unknown	5	6	13	3	9	16	7	15	9	9	9	10	13	3	6

References

1. Zheng S, Cherniack AD, Dewal N, Moffitt RA, Danilova L, Murray BA, et al. Comprehensive Pan-Genomic Characterization of Adrenocortical Carcinoma. Cancer Cell. 2016;29:723–36.

2. Ceccarelli M, Barthel FP, Malta TM, Sabedot TS, Salama SR, Murray BA, et al. Molecular Profiling Reveals Biologically Discrete Subsets and Pathways of Progression in Diffuse Glioma. Cell. 2016;164:550–63.

3. The Molecular Taxonomy of Primary Prostate Cancer. Cell. 2015;163:1011–25.

4. Linehan WM, Spellman PT, Ricketts CJ, Creighton CJ, Fei SS, Davis C, et al. Comprehensive Molecular Characterization of Papillary Renal-Cell Carcinoma. N Engl J Med. 2016;374:135–45.

5. Ciriello G, Gatza ML, Beck AH, Wilkerson MD, Rhie SK, Pastore A, et al. Comprehensive Molecular Portraits of Invasive Lobular Breast Cancer. Cell. 2015;163:506–19.

6. Genomic Classification of Cutaneous Melanoma. Cell. 2015;161:1681–96.

7. Cancer Genome Atlas Research Network. Comprehensive, integrative genomic analysis of diffuse lower-grade gliomas. New England Journal of Medicine. 2015;372:2481–98.

8. Comprehensive genomic characterization of head and neck squamous cell carcinomas. Nature. 2015;517:576–82.

9. Parfenov M, Pedamallu CS, Gehlenborg N, Freeman SS, Danilova L, Bristow CA, et al. Characterization of HPV and host genome interactions in primary head and neck cancers. Proceedings of the National Academy of Sciences. 2014;111:15544–9.

10. Integrated genomic characterization of papillary thyroid carcinoma. Cell. 2014;159:676–90.

11. Davis CF, Ricketts CJ, Wang M, Yang L, Cherniack AD, Shen H, et al. The somatic genomic landscape of chromophobe renal cell carcinoma. Cancer Cell. 2014;26:319–30.

12. Hoadley KA, Yau C, Wolf DM, Cherniack AD, Tamborero D, Ng S, et al. Multiplatform analysis of 12 cancer types reveals molecular classification within and across tissues of origin. Cell. 2014;158:929–44.

13. Cancer Genome Atlas Research N. Comprehensive molecular characterization of gastric adenocarcinoma. Nature. 2014;513:202–9.

14. Cancer Genome Atlas Research N. Comprehensive genomic characterization of squamous cell lung cancers. Nature. 2012;489:519–25.

15. Comprehensive molecular characterization of urothelial bladder carcinoma. Nature. 2014;507:315–22.

16. Comprehensive molecular characterization of human colon and rectal cancer. Nature. 2012;487:330–7.

17. Weinstein JN, Collisson EA, Mills GB, Shaw KRM, Ozenberger BA, Ellrott K, et al. The cancer genome atlas pan-cancer analysis project. Nature genetics. 2013;45:1113.

18. Yang J-Y, Yoshihara K, Tanaka K, Hatae M, Masuzaki H, Itamochi H, et al. Predicting time to ovarian carcinoma recurrence using protein markers. The Journal of clinical investigation. 2013;123:3740–50.

19. Comprehensive molecular characterization of clear cell renal cell carcinoma. Nature. 2013;499:43–9.

20. Cancer Genome Atlas Research N, Kandoth C, Schultz N, Cherniack AD, Akbani R, Liu Y, et al. Integrated genomic characterization of endometrial carcinoma. Nature. 2013;497:67–73.

21. Hakimi AA, Ostrovnaya I, Reva B, Schultz N, Chen Y-B, Gonen M, et al. Adverse outcomes in clear cell renal cell carcinoma with mutations of 3p21 epigenetic regulators BAP1 and SETD2: a report by MSKCC and the KIRC TCGA research network. Clinical Cancer Research. 2013;19:3259–67.

22. Ley TJ, Miller C, Ding L, Raphael BJ, Mungall AJ, Robertson A, et al. Genomic and epigenomic landscapes of adult de novo acute myeloid leukemia. N Engl J Med. 2013;368:2059–74.

23. Verhaak RG, Tamayo P, Yang J-Y, Hubbard D, Zhang H, Creighton CJ, et al. Prognostically relevant gene signatures of high-grade serous ovarian carcinoma. The Journal of clinical investigation. 2012;123.

24. Cancer Genome Atlas Network. Comprehensive molecular portraits of human breast tumours. Nature. 2012;490:61.

25. Larman TC, DePalma SR, Hadjipanayis AG, Protopopov A, Zhang J, Gabriel SB, et al. Spectrum of somatic mitochondrial mutations in five cancers. Proceedings of the National Academy of Sciences. 2012;109:14087–91.

26. Lee E, Iskow R, Yang L, Gokcumen O, Haseley P, Luquette LJ, et al. Landscape of somatic retrotransposition in human cancers. Science. 2012;337:967–71.

27. Creighton CJ, Hernandez-Herrera A, Jacobsen A, Levine DA, Mankoo P, Schultz N, et al. Integrated analyses of microRNAs demonstrate their widespread influence on gene expression in high-grade serous ovarian carcinoma. PLoS One. 2012;7:e34546.

28. Bolton KL, Chenevix-Trench G, Goh C, Sadetzki S, Ramus SJ, Karlan BY, et al. Association between BRCA1 and BRCA2 mutations and survival in women with invasive epithelial ovarian cancer. Jama. 2012;307:382–9.

29. Integrated genomic analyses of ovarian carcinoma. Nature. 2011;474:609–15.

30. Noushmehr H, Weisenberger DJ, Diefes K, Phillips HS, Pujara K, Berman BP, et al. Identification of a CpG island methylator phenotype that defines a distinct subgroup of glioma. Cancer Cell. 2010;17:510–22.

31. Verhaak RG, Hoadley KA, Purdom E, Wang V, Qi Y, Wilkerson MD, et al. Integrated genomic analysis identifies clinically relevant subtypes of glioblastoma characterized by abnormalities in PDGFRA, IDH1, EGFR, and NF1. Cancer Cell. 2010;17:98–110.

32. Comprehensive genomic characterization defines human glioblastoma genes and core pathways. Nature. 2008;455:1061–8.

33. Kandoth C, McLellan MD, Vandin F, Ye K, Niu B, Lu C, et al. Mutational landscape and significance across 12 major cancer types. Nature. 2013;502:333–9.

34. Tamborero D, Gonzalez-Perez A, Perez-Llamas C, Deu-Pons J, Kandoth C, Reimand J, et al. Comprehensive identification of mutational cancer driver genes across 12 tumor types. Sci Rep. 2013;3:2650.

35. Lawrence MS, Stojanov P, Mermel CH, Robinson JT, Garraway LA, Golub TR, et al. Discovery and saturation analysis of cancer genes across 21 tumour types. Nature. 2014;505:495–501.

36. Pritchard CC, Salipante SJ, Koehler K, Smith C, Scroggins S, Wood B, et al. Validation and implementation of targeted capture and sequencing for the detection of actionable mutation, copy number variation, and gene rearrangement in clinical cancer specimens. J Mol Diagn. 2014;16:56–67.

37. Ciriello G, Miller ML, Aksoy BA, Senbabaoglu Y, Schultz N, Sander C. Emerging landscape of oncogenic signatures across human cancers. Nature genetics. 2013;45:1127–33.

38. Nik-Zainal S, Davies H, Staaf J, Ramakrishna M, Glodzik D, Zou X, et al. Landscape of somatic mutations in 560 breast cancer whole-genome sequences. Nature. 2016;534:47–54.

39. Andor N, Graham TA, Jansen M, Xia LC, Aktipis CA, Petritsch C, et al. Pan-cancer analysis of the extent and consequences of intratumor heterogeneity. Nat Med. 2016;22:105–13.

40. Futreal PA, Coin L, Marshall M, Down T, Hubbard T, Wooster R, et al. A census of human cancer genes. Nat Rev Cancer. 2004;4:177–83.

41. COSMIC. COSMIC: Cancer Gene census. 2016.

42. cBioPortal. cBioPortal for Cancer Genomics. 2016.

43. cBioPortal. Stomach Adenocarcinoma (TCGA, Provisional).

44. Lonsdale J, Thomas J, Salvatore M, Phillips R, Lo E, Shad S, et al. The genotype-tissue expression (GTEx) project. Nature genetics. 2013;45:580.

45. GTEx. GTEx Portal. 2016.

46. Color Genomics. Science Powering Color - Color Genomics. 2016.

47. Rahman N. Realizing the promise of cancer predisposition genes. Nature. 2014;505:302–8.

48. Gray KA, Yates B, Seal RL, Wright MW, Bruford EA. Genenames. org: the HGNC resources in 2015. Nucleic acids research. 2014;43:D1079–85.

49. Genenames. org. The HGNC resources in 2015. 2016.

50. Maglott D, Ostell J, Pruitt KD, Tatusova T. Entrez Gene: gene-centered information at NCBI. Nucleic acids research. 2005;33:D54–8.

51. Ncbi. nl.nih.gov. NCBI Gene Search Results Entrez Gene Numbers. 2016.

52. Pei B, Sisu C, Frankish A, Howald C, Habegger L, Mu XJ, et al. The GENCODE pseudogene resource. Genome Biol. 2012;13:R51.

53. Glusman G, Yanai I, Rubin I, Lancet D. The complete human olfactory subgenome. Genome research. 2001;11:685–702.

54. HORDE. HORDE Home. 2014.

55. Khurana E, Fu Y, Chen J, Gerstein M. Interpretation of genomic variants using a unified biological network approach. PLoS Comput Biol. 2013;9:e1002886.

56. MacArthur DG, Balasubramanian S, Frankish A, Huang N, Morris J, Walter K, et al. A systematic survey of loss-of-function variants in human protein-coding genes. Science. 2012;335:823–8.

57. Arrand JR, Rymo L, Walsh JE, Björck E, Lindahl T, Griffin BE. Molecular cloning of the complete Epstein-Barr virus genome as a set of overlapping restriction endonuclease fragments. Nucleic acids research. 1981;9:2999–3014.

58. ncbi. nlm.nih.gov. Epstein-Barr virus (EBV) genome, strain B95-8. 2014.

59. nlm. nih.ncbi.gov. Helicobacter pylori ELS37, complete genome. 2014.

60. Ncbi. nl.nih.gov. Helicobacter pylori 35A, complete genome. 2014.

61. nlm. nih.ncbi.gov. Helicobacter pylori 26695 chromosome, complete genome. 2014.

62. nlm. nih.ncbi.gov. Helicobacter pylori B38 complete genome, strain B38. 2014.

63. nlm. nih.ncbi.gov. Helicobacter pylori B8 complete genome. 2014.

64. nlm. nih.ncbi.gov. Helicobacter pylori 52, complete genome. 2014.

65. nlm. nih.ncbi.gov. Helicobacter pylori 2017, complete genome. 2014.

66. nlm. nih.ncbi.gov. Helicobacter pylori HUP-B14, complete genome. 2014.

67. nlm. nih.ncbi.gov. Helicobacter pylori SouthAfrica20, complete genome. 2014.

68. Agilent Technologies. SureSelectXT2 Target Enrichment System for Illumina Paired-End Multiplexed Sequencing. 2016.

69. Illumina. HiSeq® 3000/HiSeq 4000 Sequencing Systems. 2015.

70. BROAD Institute. Best Practices for Variant Calling with the GATK | Broad Institute.

71. Team RC. R: A language and environment for statistical computing. 2013;

72. Jefferis G. Readbitmap: Simple Unified Interface to Read Bitmap Images (BMP, JPEG, PNG). R package version 0104. 2014;

73. Walker A, Braglia L. openxlsx: read, write and edit XLSX files. R package version. 2015;3.

74. Maechler M, Rousseeuw P, Struyf A, Hubert M, Hornik K. Cluster: cluster analysis basics and extensions. R package version. 2012;1:56.

75. Knaus BJ, Grünwald NJ. vcfr: a package to manipulate and visualize variant call format data in R. Molecular Ecology Resources. 2017;17:44–53.

76. Morgan M, Pages H, Obenchain V, Hayden N. Rsamtools: Binary alignment (BAM), FASTA, variant call (BCF), and tabix file import. R package version. 2016;1:677–89.

77. Rowe BLY. futile. logger: A Logging Utility for R. R package version. 2015;1.

78. Obenchain V, Lawrence M, Carey V, Gogarten S, Shannon P, Morgan M. VariantAnnotation: a Bioconductor package for exploration and annotation of genetic variants. Bioinformatics. 2014;30:2076–8.

79. Lawrence M, Huber W, Pages H, Aboyoun P, Carlson M, Gentleman R, et al. Software for computing and annotating genomic ranges. PLoS Comput Biol. 2013;9:e1003118.

80. Pagès H. BSgenome: Software infrastructure for efficient representation of full genomes and their SNPs. R package version. 2017;1:10.18129.

81. Carlson M, Maintainer B. TxDb. Hsapiens. UCSC. hg19. knownGene: Annotation package for TxDb object (s). R package version 3, 0, 0. 2015.

82. International Human Genome Sequencing Consortium. Initial sequencing and analysis of the human genome. Nature. 2001;409:860.

83. BROAD Institute. HG38 Human Reference Genome in GATK Resource Bundle. 2017.

84. GENCODE. evidence-based annotation of the human genome (GRCh37), version 19 (Ensembl 74). 2017.

85. Sibley CR, Blazquez L, Ule J. Lessons from non-canonical splicing. Nat Rev Genet. 2016;17:407–21.

86. HUGO Gene Nomenclature Committee. hgnc_complete_set.txt. 2019.

87. University of California Santa Cruz Genomics Institute. liftOver. 2017.

88. Buffalo V. Scythe. 2014.

89. Joshi NA, Fass JN. Sickle: A sliding-window, adaptive, quality-based trimming tool for FastQ files (Version 1.33). 2011.

90. Babraham Bioinformatics. FASTQC. 2017.

91. Hannon Labs. FASTX-Toolkit. 2017.

92. Li H. Aligning sequence reads, clone sequences and assembly contigs with BWA-MEM. arXiv preprint arXiv:13033997. 2013;

93. Li H. Samtools is a suite of programs…samtools, bcftools, htslib. 2017.

94. BROAD Institute. Picard. 2017.

95. McKenna A, Hanna M, Banks E, Sivachenko A, Cibulskis K, Kernytsky A, et al. The Genome Analysis Toolkit: a MapReduce framework for analyzing next-generation DNA sequencing data. Genome Res. 2010;20:1297–303.

96. DePristo MA, Banks E, Poplin R, Garimella KV, Maguire JR, Hartl C, et al. A framework for variation discovery and genotyping using next-generation DNA sequencing data. Nature genetics. 2011;43:491.

97. Van der Auwera GA, Carneiro MO, Hartl C, Poplin R, Del Angel G, Levy-Moonshine A, et al. From FastQ data to high-confidence variant calls: the genome analysis toolkit best practices pipeline. Current protocols in bioinformatics. 2013;43:11.10. 1-11.10. 33.

98. Quinlan AR, Hall IM. BEDTools: a flexible suite of utilities for comparing genomic features. Bioinformatics. 2010;26:841–2.

99. Garcia-Alcalde F, Okonechnikov K, Carbonell J, Cruz LM, Gotz S, Tarazona S, et al. Qualimap: evaluating next-generation sequencing alignment data. Bioinformatics. 2012;28:2678–9.

100. Karczewski KJ, Francioli LC, Tiao G, Cummings BB, Alföldi J, Wang Q, et al. Variation across 141,456 human exomes and genomes reveals the spectrum of loss-of-function intolerance across human protein-coding genes. bioRxiv. 2019;531210.

101. Niu B, Ye K, Zhang Q, Lu C, Xie M, McLellan MD, et al. MSIsensor: microsatellite instability detection using paired tumor-normal sequence data. Bioinformatics. 2014;30:1015–6.

102. BROAD Institute. Mutect2. 2017.

103. BROAD Institute. GATK | Doc #11136 | (How to) Call somatic mutations using GATK Mutect2 (Deprecated).

104. Josephidou M, Lynch AG, Tavare S. multiSNV: a probabilistic approach for improving detection of somatic point mutations from multiple related tumour samples. Nucleic Acids Res. 2015;43:e61.

105. Benson G. Tandem repeats finder: a program to analyze DNA sequences. Nucleic acids research. 1999;27:573–80.

106. Rosenbloom KR, Armstrong J, Barber GP, Casper J, Clawson H, Diekhans M, et al. The UCSC Genome Browser database: 2015 update. Nucleic Acids Res. 2015;43:D670-81.

107. University of California Santa Cruz Genome Bioinformatics Group. UCSC Genome Browser.

108. Karolchik D, Hinrichs AS, Furey TS, Roskin KM, Sugnet CW, Haussler D, et al. The UCSC Table Browser data retrieval tool. Nucleic acids research. 2004;32:D493–6.

109. Tyner C, Barber GP, Casper J, Clawson H, Diekhans M, Eisenhart C, et al. The UCSC Genome Browser database: 2017 update. Nucleic acids research. 2017;45:D626-d634.

110. Wang K, Li M, Hakonarson H. ANNOVAR: functional annotation of genetic variants from high-throughput sequencing data. Nucleic Acids Res. 2010;38:e164.

111. Genomes Project Consortium. A global reference for human genetic variation. Nature. 2015;526:68.

112. National Center for Biotechnology Information NL of M. Database of single nucleotide polymorphisms (dbSNP). 1998;

113. Tate JG, Bamford S, Jubb HC, Sondka Z, Beare DM, Bindal N, et al. COSMIC: the catalogue of somatic mutations in cancer. Nucleic acids research. 2018;47:D941–7.

114. Landrum MJ, Lee JM, Benson M, Brown GR, Chao C, Chitipiralla S, et al. ClinVar: improving access to variant interpretations and supporting evidence. Nucleic acids research. 2017;46:D1062–7.

115. Lek M, Karczewski KJ, Minikel EV, Samocha KE, Banks E, Fennell T, et al. Analysis of protein-coding genetic variation in 60,706 humans. Nature. 2016;536:285–91.

116. McCarthy S, Das S, Kretzschmar W, Delaneau O, Wood AR, Teumer A, et al. A reference panel of 64,976 haplotypes for genotype imputation. Nature genetics. 2016;48:1279.

117. Riester M, Singh AP, Brannon AR, Yu K, Campbell CD, Chiang DY, et al. PureCN: copy number calling and SNV classification using targeted short read sequencing. Source Code Biol Med. 2016;11:13.

118. Derrien T, Estellé J, Sola SM, Knowles DG, Raineri E, Guigó R, et al. Fast computation and applications of genome mappability. PLoS One. 2012;7:e30377.

119. Paolo Ribeca. gem-mappability.04-05-2018.tar.xz. 2018.

120. Zack TI, Schumacher SE, Carter SL, Cherniack AD, Saksena G, Tabak B, et al. Pan-cancer patterns of somatic copy number alteration. Nat Genet. 2013;45:1134–40.

121. Chakravarty D, Gao J, Phillips S, Kundra R, Zhang H, Wang J, et al. OncoKB: a precision oncology knowledge base. JCO precision oncology. 2017;1:1–16.

122. OncoKB. OncoKB Precision Oncology Knowledge Base. 2020.

123. Ichikawa H, Nagahashi M, Shimada Y, Hanyu T, Ishikawa T, Kameyama H, et al. Actionable gene-based classification toward precision medicine in gastric cancer. Genome Med. 2017;9:93.

124. Alexandrov LB, Nik-Zainal S, Wedge DC, Aparicio SA, Behjati S, Biankin AV, et al. Signatures of mutational processes in human cancer. Nature. 2013;500:415–21.

125. Alexandrov LB, Nik-Zainal S, Wedge DC, Campbell PJ, Stratton MR. Deciphering signatures of mutational processes operative in human cancer. Cell Rep. 2013;3:246–59.

126. Alexandrov LB, Nik-Zainal S, Siu HC, Leung SY, Stratton MR. A mutational signature in gastric cancer suggests therapeutic strategies. Nat Commun. 2015;6:8683.

127. Alexandrov LB, Kim J, Haradhvala NJ, Huang MN, Tian Ng AW, Wu Y, et al. The repertoire of mutational signatures in human cancer. Nature. 2020;578:94–101.

128. Rosenthal R, McGranahan N, Herrero J, Taylor BS, Swanton C. DeconstructSigs: delineating mutational processes in single tumors distinguishes DNA repair deficiencies and patterns of carcinoma evolution. Genome Biol. 2016;17:31.

129. Paradis E, Schliep K. ape 5.0: an environment for modern phylogenetics and evolutionary analyses in R. Bioinformatics. 2018;35:526–8.

130. Harmon LJ, Weir JT, Brock CD, Glor RE, Challenger W. GEIGER: investigating evolutionary radiations. Bioinformatics. 2007;24:129–31.

131. Desper R, Gascuel O. Fast and accurate phylogeny reconstruction algorithms based on the minimum-evolution principle. J Comput Biol. 2002;9:687–705.
